# Systematic analysis of secreted proteins reveals synergism between IL6 and other proteins in soft agar growth of MCF10A cells

**DOI:** 10.1186/2045-3701-1-13

**Published:** 2011-03-25

**Authors:** Sofie C Van Huffel, Jill M Tham, XiaoQian Zhang, KohPang Lim, ChunXuan Yang, YikLoo Tan, Felicia Ong, Ian Lee, WanJin Hong

**Affiliations:** 1 Cancer and Developmental Cell Biology Division (CDCBD), Institute of Molecular and Cell Biology, A-star, 61 Biopolis Drive, Proteos, 138673, Singapore; 2 Genome Institute of Singapore, A-star, 60 Biopolis Street, #02-01, Genome, 138672, Singapore

## Abstract

**Introduction:**

Breast cancer, the most common malignancy in women, still holds many secrets. The causes for non-hereditary breast cancer are still unknown. To elucidate any role for circulating naturally secreted proteins, a screen of secreted proteins' influence of MCF10A cell anchorage independent growth was set up.

**Methods:**

To systematically screen secreted proteins for their capacity to transform mammalian breast epithelial cells, a soft agar screen of MCF10A cells was performed using a library of ~ 470 secreted proteins. A high concentration of infecting viral particles was used to obtain multiple infections in individual cells to specifically study the combined effect of multiple secreted proteins.

**Results:**

Several known breast cancer factors, such as Wnt, FGF and IL were retained, as well as factors that were previously unknown to have a role in breast cancer, such as paraoxonase 1 and fibroblast growth factor binding protein 2. Additionally, a combinatory role of Interleukin 6 with other factors in MCF10A anchorage-independent growth is demonstrated.

**Conclusion:**

The transforming effect of combinations of IL6 with other secreted proteins allows studying the transformation of mammary epithelial cells *in vitro*, and may also have implications in *in vivo *studies where secreted proteins are upregulated or overexpressed.

## Introduction

Breast cancer is the most common malignancy in women, accounting for 1/3 of all cancers in women and the highest cancer-related mortality in women. While many breast cancer oncogenes have been identified, the causes for the majority of sporadic breast cancers are unknown.

Studies with Mouse Mammary tumor Virus (MMTV) in mice have indicated that enhanced expression of a number of secreted proteins, such as Wingless-type MMTV integration site family (Wnt), Fibroblast growth factor (FGF) and R-spondin (RSPO), are associated with the development of breast cancer [1-7]. However, when overexpressed in mammary epithelial cells, these proteins do not transform cells. Also, mice transgenic for Wnt or FGF proteins develop breast cancer in a stochastic manner, indicating that a second event is necessary for breast cancer development [1,8]. Taken together, these findings indicate that while these secreted factors are important contributing factors, individually, they are not sufficient for full transformation of breast epithelial cells.

It has also been shown that cytokines can have both a stimulatory or inhibitory effect on breast cancer cells, depending on their relative concentrations and the presence of other factors in the microenvironment [9]. This indicates that the cooperative or antagonistic effects of multiple secreted factors may be more decisive than the individual factors in determining cellular behavior.

We have set out to investigate the co-operative action of secreted proteins in the transformation of breast epithelial cells. We have constructed an expression library of 470 secreted proteins in a retroviral vector, and used it to infect human breast epithelial cells at high multiplicity of infection (MOI), with the goal to achieve multiply infected cells, expressing combinations of secreted factors. These cells were selected by growth in soft agar, and the infecting cDNA's were recovered. Analysis of about 300 colonies revealed that it is indeed possible to obtain multiple infection of MCF10A cells through retroviral infection. The cDNA's recovered from the selected cells reveal a high recurrence of genes that have previously been implicated in breast cancer development, such as Wnt and FGF proteins, while at the same time revealing high recurrences of genes that had previously not been directly linked to breast cancer development, such as FGF-binding proteins and paraoxonases. Furthermore, the high incidence of multiple infections in the selected colonies indicate that multiple factors are needed for successful anchorage-independent growth of MCF10A cells. We demonstrate that particularly interleukin 6, when combined with other factors, but not alone, is effectively driving anchorage independent growth in MCF10A cells.

## Materials and methods

### 1. Cell lines

MCF10A cells were obtained from American Type Culture Collection and grown in Dulbecco's modified Eagle's medium supplemented with 5% horse serum, 20 ng/ml epidermal growth factor, 0.5 μg/ml hydrocortisone, 100 mg/ml cholera toxin, 10 μg/ml insulin and penicillin/streptomycin. The amphotropic Phoenix packaging cell line was obtained from the Nolan Laboratory, Stanford University and grown in DME medium, supplemented with 10% Fetal Calf Serum.

### 2. Cloning of library

A total of 469 cDNA's were cloned into either pBABE-puro, or a slightly modified pBABE-puro, pBABE-I, retroviral vectors (see additional file 1: figure S1). pBABE-I was created by replacing the SV40 promoter in pBABE-puro with the PGK promoter by PCR from the pSIREN vector (Clontech). The PGK promoter fragment was amplified by PCR with primers forward: GAGGGATCCGAATTCCACCATGGCTGACAGATCTTAATGAGTCGACAATTGTACCGGTAGGGGAGGC; and reverse: AGAGTTCTTGCAGCTCGGTGAC; and cloned into the BamHI-BsiWI cut pBABE-puro. The primers contain restriction enzyme recognition sites (underlined) for an altered MCS in pBABE-I. No difference in production of viral particles by either vector has been observed. About half of the clones were cloned in pBABE-puro, and about half in pBABE-I. Most cDNA's were cloned by PCR from the MegaMan Human Transcriptome Library (Stratagene), or were obtained by RT-PCR from tissue-specific mRNA (purchased from Clontech). Others were cloned by PCR from EST clones that were obtained via RZPD (Germany) or the IMAGE consortium (via ATCC). Before the ATG codon of the clones, a KOZAK sequence was inserted. All clones were verified by full-length sequencing.

### 3. Soft agar screen

The library was subdivided in 13 random groups of about 37 clones, and transfected in the AMPHO cell line using Effectene (Qiagen). Forty-eight hours later, virus-containing supernatants from all groups were combined (about 790 ml was recovered), filtered (0.45 μM filter; Millipore) and concentrated by spinning in SA600 rotor at 15000 RPM for 2 hours in a Sorvall centrifuge. The viral pellets were resuspended in a final volume of 90 ml. This medium was supplemented with 5 μg/ml polybrene (Sigma-Aldrich), and evenly distributed over 6 15-cm dishes of MCF10A cells that had been seeded at 3 × 10^6 ^cell/dish 24 hours before.

After 24 hours, the infected MCF10A cells were trypsinized, counted, and seeded in puromycin (1 μg/ml) containing soft agar (2 × 10^6 ^cell/15 cm dish). The dishes were maintained for four weeks, and 15 ml of medium with puromycin was added once per week. Many differently sized colonies could be observed. A total of 400 larger colonies were picked and plated in normal puromycin-containing medium and allowed to grow and expand. About 300 colonies were found to be viable. Total DNA was purified from 10^6 ^cells of each expanded colony using the FlexiGene DNA kit (Qiagen). Three different PCR reactions were performed with each genomic DNA samples, one with G3PDH primers to verify DNA quality, one with a set of pBABE-puro (Primers: For: ctaagcctccgcctcctcttcttcc; Rev: gcctcccctacccggtagaattgtc) and the last with pBABE-I (Primers: For: ctaagcctccgcctcctcttcttcc; Rev: ggactttccacacctggttgctgac) specific primers, respectively. All PCR products were purified from agarose gels and subjected to direct sequencing with primer gcctcctcttcttccatccg. Sequencing results were subjected to BLAST searches on the NCBI webservice to identify the inserts.

### 4. Soft agar setups (small scale)

AMPHO cells were transfected using FuGene 6 (Roche) or Effectene (Qiagen) according to manufacturer recommendations. The obtained viral supernatant was filtered and used to infect 6-well of MCF10A cells. Three changes of the viral supernatant was used over a period 48 hours. When combinations of different protein interactions were required, individual retroviral constructs were transfected, and viral supernatants were combined at the time of infection. Infected cells underwent puromycin-selection and were grown for 3-5 days, trypsinized, counted and seeded in triplicates of soft agar base at 20,000 cells/well in 6-well dishes. Fresh Medium was added twice per week with the old medium being removed once during the week. After 4 weeks, colonies were stained with Thiazolyl Blue Tetrazolium Bromide for 4 hours at room temperature, destained multiple times with water, and then analyzed.

## Results and Discussion

### 1. Construction of library

To construct the secretomics library, we selected candidate proteins on basis of reported secretion, predicted secretory domains or signals, or homology to known secreted proteins. Most of the proteins in our library are 500 or less amino acids in size, so as to allow for efficient infection and expression in a retroviral system. At the time of the experiments described in this report, our library contained 467 different proteins. Our library contained both individual proteins, and multiple members of protein families, such as 16 different Wnt proteins, 22 FGF proteins, 35 interleukins, and 15 kalikreins (see additional file 2: table S1). The library was constructed in the selectable retroviral vector pBABE to allow for wide application.

### 2. Infection of MCF10A cells

**Figure 1:**
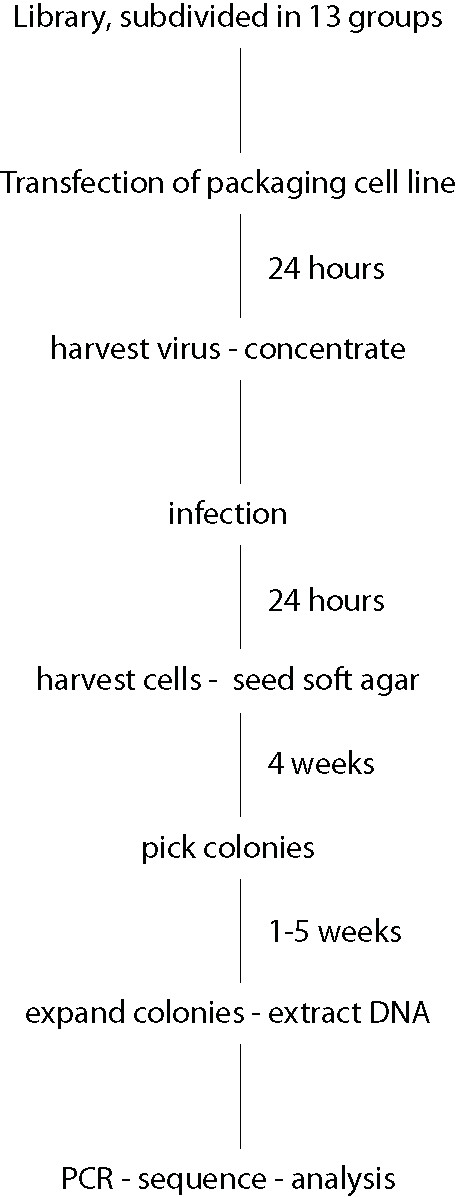
**Flow-chart of screen procedures**.

In order to study the combinatory effect of multiple secreted factors on a single cell, we infected the cells with a mixture of retroviral particles at a high concentration. A Flow-chart of the screen process is shown in figure 1. We subdivided the library in 13 subgroups, which were used to transfect the amphotropic packaging cell line. To ensure complete random infection, the retroviral particles obtained from the supernatant of the transfected subgroups were combined, concentrated by centrifugation, and applied to the MCF10A cells. The infected cells were seeded in soft agar, where wild type MCF10A cells are unable to grow, and selected for 4 weeks. About 400 of the formed colonies were extracted from the soft agar and grown in MCF10A medium with puromycin under normal growth conditions.

About 300 of these colonies grew successfully and were expanded, and genomic DNA was extracted. The incorporated clonal DNAs were amplified from the total genomic DNA by PCR with retroviral vector-specific primers, purified from the agarose gel, and then sequenced. The sequences were scanned for the recognizable vector-specific sequences and as well as subjected to BLAST analysis.

**Figure 2:**
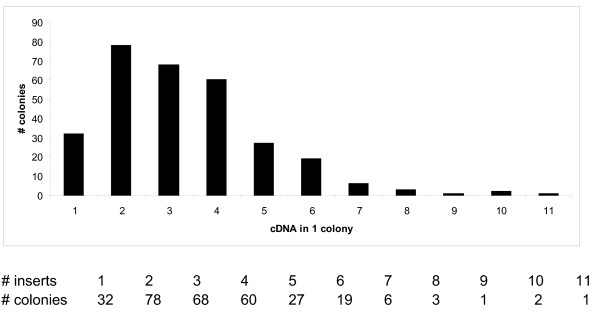
**Distribution of viral infections**. The number of colonies from which the same number of cDNA inserts were recovered shows multiple infection took place in the screen.

Analysis of the recombinant DNAs that were recovered from the selected soft agar colonies revealed that both single and multiple infections had occurred. As shown in figure 2, multiple infections (up to 11 inserts in one colony) of different DNA insertions were achieved in this screen. The majority of recovered colonies (about 70%; 206 out of 297 colonies) contained combinations of 2 to 4 DNA inserts. Only about 10% of colonies (32 out of 297) contained a single DNA insert, indicating that the over expression of a single insertion may be less efficient to promote anchorage independent growth in MCF10A.

### 3. Screen analysis

The full list of recovered inserts is listed in the additional tables. Additional file 3: Table S2 gives a listing of all inserts recovered from the colonies while Additional file 4: Table S3 gives the same listing classified by the family of proteins. Additional file 5: Table S4 gives the actual inserts identified from each colony picked.

The top inserts are listed, by recurrence of individual insertions with the highest frequency in Table 1 and by recurrence of members of a protein family in Table 2.

**Table 1:** List of DNA inserts from screen colonies

**total inserts**	**% of all inserts**	**protein**	**Protein family**	**Description**	**Role in Breast cancer**
**68**	6.9%	**WFDC13**	WFDC	WAP Four-Disulfide Core Domain 13	no
**36**	3.6%	**PON1**	PON	Paraoxonase 1	no
**31**	3.1%	**Wnt3**	Wnt	Wingless-type MMTV Integration Site Family, Member 3	[7]
**18**	1.8%	**FGFBP2**	FGFBP	Fibroblast Growth Factor Binding Protein 2	no
**15**	1.5%	**IL6**	Interleukin	Interleukin 6 (Interferon, Beta 2)	[10]
**15**	1.5%	**FGF14**	FGF	Fibroblast Growth Factor 14	no
**14**	1.4%	**SAA1**	SAA	Serum Amyloid A1	[24]
**14**	1.4%	**PTHLH**	PTHLH	Parathyroid Hormone-Like Hormone	[23]
**13**	1.3%	**FGF10**	FGF	Fibroblast Growth Factor 10	[6]
**12**	1.2%	**LYNX1**	Ly-6	Ly6/Neurotoxin 1	No
**12**	1.2%	**IL21**	Interleukin	Interleukin 21	Refs in [38]
**11**	1.1%	**IL22**	Interleukin	Interleukin 22	[16]
**10**	1.0%	**WFDC14**	WFDC	Peptidase Inhibitor 3, Skin-Derived (SKALP)	no
**10**	1.0%	**SUMF1**	SUMF	Sulfatase Modifying Factor 1	no
**10**	1.0%	**FGF1**	FGF	Fibroblast Growth Factor 1 (acidic)	[22]

The most recurrent DNA inserts recovered from screen colonies are listed, with indication of the protein family they belong to, and publications linking the proteins to breast cancer development.

**Table 2:** List of protein families recovered from screen colonies

**total inserts**	**Total colonies**	**% of all colonies**	**Protein family**	
**97**	**83**	**27.9%**	Interleukin	Interleukin
**97**	**88**	**29.5%**	WFDC	WAP Four-Disulfide Core Domain
**78**	**72**	**24.2%**	FGF	Fibroblast Growth Factor
**54**	**54**	**18.1%**	Wnt	Wingless-type MMTV Integration Site Family
**40**	**39**	**13.1%**	PON	Paraoxonase
**35**	**34**	**11.4%**	KLK	Kalikrein
**33**	**28**	**9.4%**	TGFB	Transforming Growth Factor
**21**	**19**	**6.4%**	CXCL	CXC Chemokine Ligand
**21**	**21**	**7.0%**	DEF	Defensin
**21**	**21**	**7.0%**	FGFBP	Fibroblast Growth Factor Binding Protein
**26**	**26**	**8.7%**	TIMP	Tissue Inhibitor of Metalloproteinase
**19**	**19**	**6.4%**	CCL	CC Chemokine Ligand
**19**	**19**	**6.4%**	IGFBP	Insulin-like Growth Factor Binding Protein
**18**	**18**	**6.0%**	RSPO	R-Spondin
**16**	**15**	**5.0%**	DKK	Dickkopf
**15**	**15**	**5.0%**	SAA	Serum Amyloid
**14**	**14**	**4.7%**	PTHLH	Parathyroid Hormone-Like Hormone
**14**	**14**	**4.7%**	SFTP	Surfactant Protein
**14**	**14**	**4.7%**	WISP	Connective Tissue Growth Factor
**13**	**13**	**4.4%**	APO	Apolipoprotein
**13**	**13**	**4.4%**	Ly-6	Ly6/uPAR
**13**	**13**	**4.4%**	PGLYRP	Peptidoglycan Recognition Protein
**12**	**12**	**4.0%**	MASP	MBL-associated Serine Protease
**10**	**10**	**3.4%**	FAM3	Family with Sequence Similarity 3
**10**	**10**	**3.4%**	IFN	Interferon
**10**	**10**	**3.4%**	SPARC	Secreted Protein Acidic and Rich in Cysteine
**10**	**10**	**3.4%**	SUMF	Sulfatase Modifying Factor

The DNA recovered from the screen colonies was grouped in protein families, and the most prevalent protein families are listed here. As multiple protein family members may co-occur in a single colony, the number of colonies may be less than the number of inserts where the protein family is represented. Proteins that have no family members in this screen are listed as individual proteins

Several of the highly recurring proteins in our screen were previously shown to be involved in breast cancer development.

Interleukins as a family of proteins are widely represented in this screen. With 97 inserts, spread over 83 colonies, interleukins are present in just under one third of the colonies. Interleukin 6 (16 inserts), IL21 (12 inserts), IL22 (11 inserts) and IL27 (9 inserts) are the most recurring interleukins. Serum levels of IL6 have been found to be elevated in breast cancer patients [10], and pretreatment IL6 serum levels can predict treatment outcome and prognosis [11,12].

Furthermore, autocrine IL-6 expression causes multidrug resistance in breast cancer cells [13], and IL-6 enhances mammosphere formation and self-renewal [14]. IL21-expressing breast adenocarcinoma cells did not have growth characteristics different from parental cells, but could not efficiently form tumors in mice [15], and protected the mice from subsequent challenges with the parental adenocarcinoma cells, indicating an immunological response. IL22 has been reported to inhibit proliferation of breast adenocarcinoma cells, both *in vivo *and *in vitro *[16]. IL27 has not been implicated in breast cancer, but has a potential role in other tumor types [17,18].

IL6 inhibition and IL21, among other interleukins, are currently being studied in clinical trials for different tumor types [19,20].

Wnt3, with 31 recurrences in this screen, and its close homologue Wnt3a were also highly recurrent in a mouse study on breast cancer development by MMTV infection [7]. Remarkable is that Wnt1, which was the first Wnt gene to be implicated in breast cancer [3] was only recovered in 1 colony in our screen.

Our study also reveals high recurrence of FGF14, FGF10 and FGF1. MMTV studies in mice have implicated FGF3, FGF4, FGF8 and FGF10 (reviewed in: [6,21]) in mouse breast cancer development. Other FGF's have not been directly implicated in breast cancer, but the role of FGF receptor 2 (FGFR2) indirectly implicates FGF1, FGF2, FGF3, FGF4, FGF6, FGF7, FGF9, FGF10, FGF16, FGF20 and FGF22 [22]. While several FGF proteins had been previously implicated in breast cancer development, we found FGF14, previously not implicated in breast cancer development, to be the highest recurring FGF in our screen. Equally remarkable is that, while FGF3, FGF4 and FGF8 had been implicated before, they did not feature high in our screen, with FGF14, FGF10 and FGF1 being high up in our screen.

Parathyroid hormone like hormone (PTHLH, also Parathyroid hormone related protein, PTHrP), recurring 14 times in our screen, is elevated in 60 to 90% of breast cancers, and is implicated in breast cancer metastasis to bone [23]. Serum amyloid proteins (SAA1/2) are represented by SAA1 in our screen, and SAA1 present in 14 of our colonies. Serum levels of this acute-phase protein are increased in cancer patients, including breast cancer patients [24], with an indication that higher SAA levels in breast cancer patients represent more metastasis and poorer prognosis.

WAP Four-Disulfide Core Domain (WFDC) proteins have not been thoroughly studied. Many WFDC coding genes are clustered on 20q12-13.1, a region that is amplified in several cancers, including breast cancer [25,26]. Four WFDC proteins have been indicated as candidate cancer biomarkers (WFDC14 (elafin), WFDC4 (SLPI), WFDC2 and WFDC1) [27]. WFDC13, the most prevalent insert in our screen has not been studied before. Other prevalent WFDC proteins in our screen are WFDC14, WFDC4 and WFDC11.

The second-most prevalent protein in our screen is paraoxonase1 (PON1), a High density lipoprotein-associated enzyme, naturally secreted by the liver, that shows protective properties in cardiovascular disease and organophosphate poisoning [28]. Few reports suggest a possible link between PON1 and breast cancer: the L55M single nucleotide polymorphism in PON1 has been associated with increased risk of breast cancer in post menopausal women [29], while another SNP (Q192R) had no effect in one study [29], but showed a decreased breast cancer risk in another study [30]. The PON1 clone in our library has L on pos 55 and Q on pos 192. PON1 expression is reported to be upregulated by estrogen receptor alpha (ERα) [31], and independently by resveratrol, a substance believed to be the main active ingredient in the cardio-protective effects of red wine, and which is also activating the ER receptor [31].

Fibroblast growth factor binding protein 2 or FGFBP2 (also termed Ksp37, killer specific secretory protein of 37 kDa) is secreted by Th1-type CD4+ lymphocytes with cytotoxic potential [32]. Increased FGFBP2 expression has been correlated with longer survival in glioblastoma [33]. Other links to cancer have not been published. Family member FGF-BP1 however has been implicated in breast cancer.

Lynx1 is a member of the lymphocyte Antigen 6 (Ly-6) superfamily of cysteine-rich proteins that are mostly GPI anchored to the cell membrane. Only 3 proteins in the family are secreted, and Lynx-1 (or SLURP-2) has been described to be upregulated in Psoriasis vulgaris [34], while another non-secreted family member, Ly-6K, was identified as a biomarker for breast cancer [35].

The sulfatase modifying factor 1 (SUMF1) that is present in 10 of our colonies has previously never been described as involved in any types of tumors. It is essential for the post-translational modification of sulfatases, and deficiency results in an autosomal recessive disorder [36].

### 4. Reconstructive analysis

As had been previously reported for a number of the proteins tested in this screen, the infection of MCF10A cells with single viral vectors led to no or very weak growth in soft agar (data not shown).

**Figure 3:**
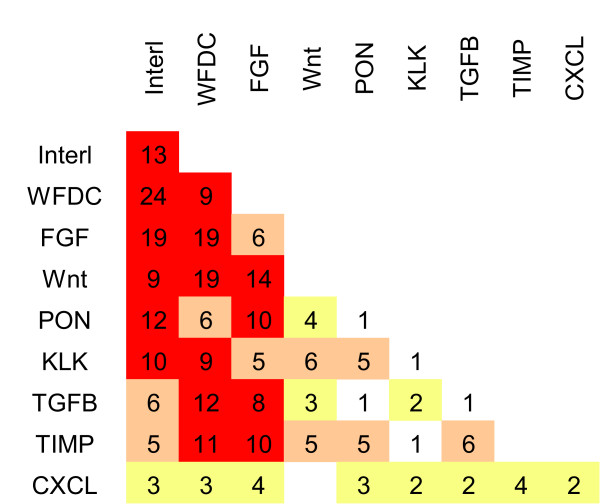
**Analysis of combinations recovered from the screen**. The number of times proteins from the indicated protein families occur in a single colony. Yellow boxes indicate numbers from 2-4, orange indicate 5-7, and red indicate more than 7 colonies containing the indicated combination.

In performing this screen, MCF10A cells were purposefully infected with concentrated viral particles, in order to achieve multiple infection of the cells. In this way, the importance of the combination of different factors on the growth of MCF10A cells in soft agar could be assessed. A schema of some combinations of factors is provided in figure 3, where the more prevalent recurring combinations are indicated in red. While we created viral particles by transfecting 13 distinct subgroups, the pooling and concentration of the viral particles rendered a random distribution in infection. This was observed by the fact that the factors found in the soft agar colonies did not return with a clear correlation to the distribution in the 13 subgroups.

**Figure 4:**
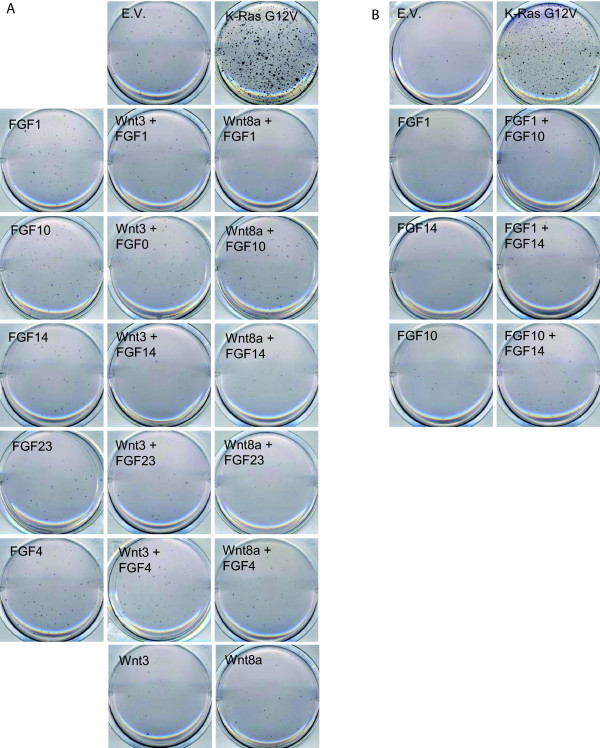
**Combinations of FGF, or FGF with Wnt do not induce growth in soft agar**. MCF10A cells were infected with the indicated cDNA's, and seeded in soft agar conditions. After four weeks of growth, wells were stained with MTT and destained with water. One of three replicates is shown.

It had been previously observed in MMTV-mouse studies that FGF and Wnt genes may co-operate in breast cancer development. Mice that are transgenic for a Wnt protein predominantly overexpressed FGF genes in MMTV-induced breast tumors, and *vice versa *[1,2,37]. A total of 14 of the colonies in our screen contained combinations of Wnt and FGF family members, suggesting that FGF-Wnt co-operation in transformation of human mammalian cells may also occur. However, when any of the top 5 FGFs were co-infected with Wnt 3 or Wnt8a in MCF10A cells, we did not observe strong growth of these cells in soft agar (figure 4A), indicating that on a cellular level, the FGF-Wnt combination is not sufficient to drive efficient transformation. Wnt9a could not be tested in this assay, as infection of Wnt9a reduced the survival of the MCF10A cells, not allowing the soft agar assay.

Six of the colonies in the present screen contained a combination of two FGF proteins. However, co-infection of two FGF proteins into MCF10A cells did not lead to significant growth in soft agar (figure 4B).

**Figure 5:**
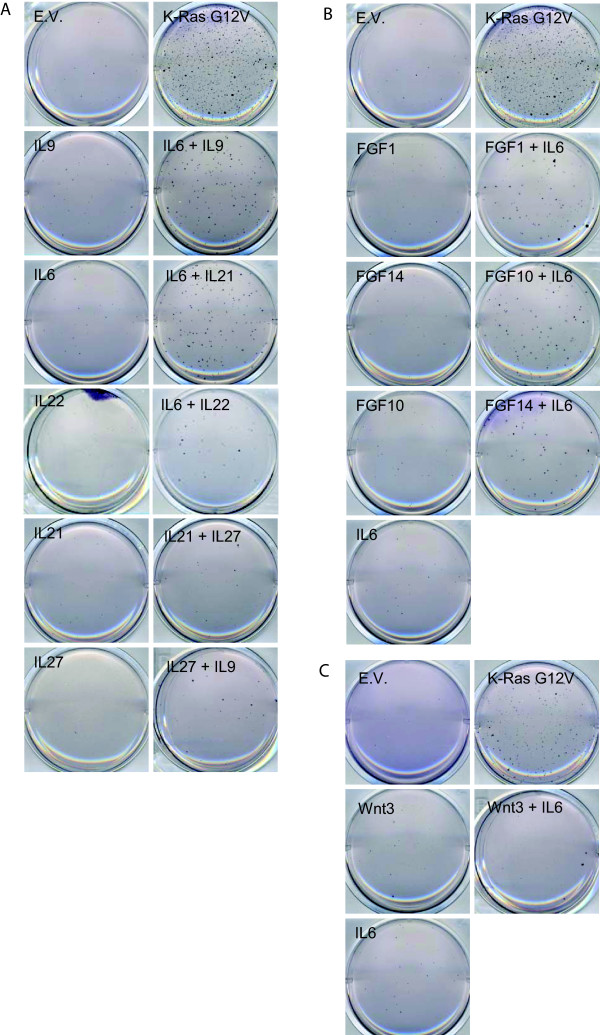
**Combinations of Interleukins, or interleukin 6 with FGF induce soft agar growth, while IL6 combined with Wnt does not**. MCF10A cells were infected with the indicated cDNA's, and seeded in soft agar conditions. After four weeks of growth, wells were stained with MTT and destained with water. One of three replicates is shown.

In 13 colonies, multiple interleukin family members were observed to be present. Infection of combinations of interleukins in MCF10A cells, lead to the observation that Interleukin 6 (IL6), when combined with IL9 or IL21 efficiently drives soft agar growth in MCF10A cells. Other combinations, such as IL6 with IL22, IL21 with IL27; or IL9 with IL27, did result in some soft agar growth, but to a much lesser extent than IL6-containing combinations (figure 5A).

Another prevalent combination in the screen was interleukins and FGF proteins (19 colonies). When these combinations were tested in MCF10A cells, IL6 in combination with FGF1, FGF10 or FGF14, proved to efficiently drive these cells to grow in soft agar, while the FGF alone, or IL6 alone, could not do so (Figure 5B). This is however in contrast to Wnt proteins, which do not show any increased growth in soft agar after co-infection with IL6 (Figure 5C).

**Figure 6:**
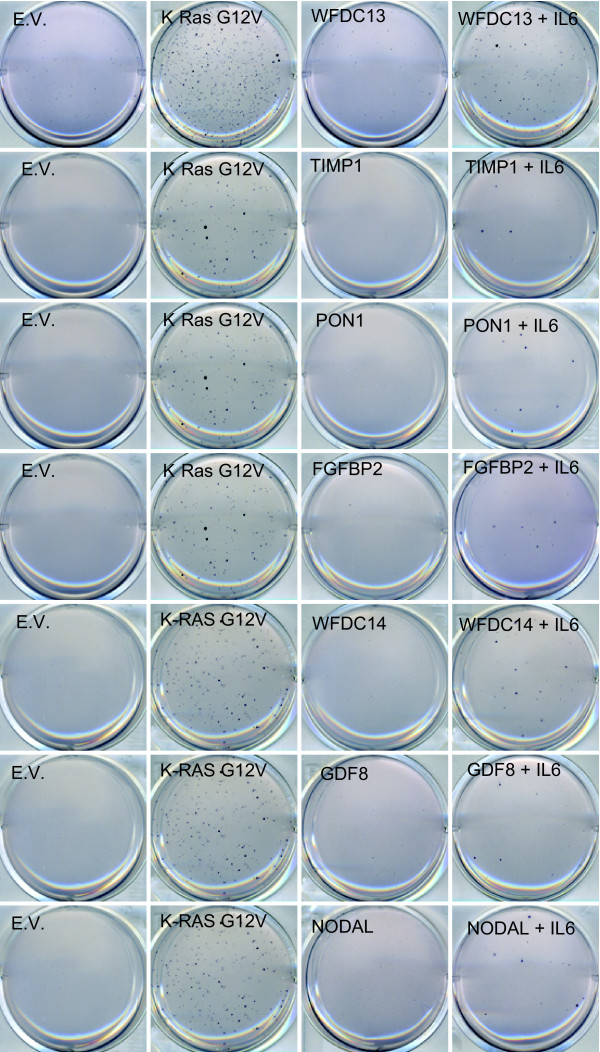
**Combinations of IL6 with other proteins results in soft agar growth**. MCF10A cells were infected with the indicated cDNA's, and seeded in soft agar conditions. After four weeks of growth, wells were stained with MTT and destained with water. One of three replicates is shown.

Seeing how interleukins and fibroblast growth factors both show increased growth in soft agar after co-infection with IL6, other prevalent proteins from our screen were tested in combination with IL6, and grown in soft agar. As shown in figure 6, MCF10A cells infected with combinations of IL6 with many of the top inserts of the screen could grow in soft agar to some extent. While soft agar growth of the tested combinations was not really robust, it was significantly stronger than soft agar growth induced by the individual proteins.

## Conclusions

With this screen, the combinatory effect of secreted factors on breast cancer development was assessed, through their action on anchorage-independent growth of MCF10A cells. While screens looking for individual proteins involved in tumor development have been described many times, this is the first screen looking for combinatory action of proteins. In the living organism, it is understood that every cell and tissue is constantly surrounded by a mix of hormones, secreted factors, as well as factors in the extracellular matrix. A different composition of this environment can seriously affect the signaling events within the cells or tissue, and influence the growth and other characteristics of the cells and tissue.

One of the benefits of this screen is also that it screened a large number of proteins that were categorized in familial clusters (e.g. 22 FGF proteins, 16 Wnt proteins, 35 interleukins). This brought more obscure protein family members in the spotlight than conventional research had done so far. Often, the more recently discovered members of protein families remain obscure for longer periods of time, as their functions are often extrapolated from more researched close family members.

Alternative screens have utilized expression profiles or 2D-gel electrophoresis combined with sequencing to directly identify proteins expressed or secreted in specific tissues. While these assays are definitely valid, they usually will have problems picking up subtle differences in expression, or proteins that are secreted at low levels. Furthermore, the validation of these proteins in *in vitro *assays may be hampered by the fact that alone, these proteins may not induce the phenotype they were screened for, and specific combinations of factors may not be recognized.

Proteins that had previously been implicated in breast cancer development, such as fibroblast growth factors and wnt proteins, were also highly recurrent in the present screen. As had been shown before in mouse studies, individual MMTV insertions appear to be sufficient for tumor formation in mouse, but in MCF10A cells, overexpression of the corresponding single protein cannot induce strong soft agar growth. While studies with MMTV in mouse indicated a potential combinatory role for Wnt and FGF [1,2,37] in breast cancer development, in the human cell line MCF10A, these two factors did not induce anchorage-independent growth. This apparent discrepancy could be due to the difference in species, but also to the in vivo nature of the mouse experiments, and the in vitro nature of the present screen. In mouse, natural secreted proteins present in the tissue could further influence the cancer development, while in the controlled environment of tissue culture, this is not the case.

A surprising combination of proteins that did induce anchorage independent growth in MCF10A cells were interleukins, which, when combined with other factors, often greatly enhanced soft agar growth. Interleukin 6, when co-expressed with other proteins prevalent in our screen, induced growth in soft agar, albeit to varying degrees, depending on the co-expressed protein. The most growth-inducing combination we detected were combinations between interleukins, such as IL6-IL21, or IL6-IL9. The combination between IL6 and FGF1, FGF10 or FGF14 also resulted in strong growth of MCF10A in soft agar. Only a few of the tested combinations with IL6 did not significantly induce soft agar growth of MCF10A. Among these was Wnt3, which, when expressed alone, already has a limited soft agar growth-inducing potential.

We have discussed above what combinations of highly expressing proteins promote anchorage independent growth in soft agar. However, some of the combinations found in the initial screen did not induce colony formation in the validation studies. For this we should consider other factors such as over-expression (both relative and absolute) and the influence of proteins secreted by surrounding cells during the screening process.

The findings in the present study can have important implications, even for seemingly unrelated research fields. For example, paraoxonase 1, overexpression of which had hitherto not been associated with breast cancer, has been mainly studied for its beneficial effect in organophosphate poisoning. Using this protein in prevention of poisoning (e.g. in chemical warfare) may have serious implications, such as breast tumor development, should at the time of high PON1 levels, IL6 levels also be raised.

The results of this screen show that it is possible to find combinatory effects of proteins in a cell-based screen, and that such combinatory effects can be very different from the individual proteins' effects. Further analysis will have to be done to examine which signal transduction pathways are involved in the stimulatory effect IL6 has with other proteins in anchorage-independent growth of mammary epithelial cells.

## List of abbreviations

(FGF): Fibroblast growth factor; (FGFBP2): Fibroblast growth factor binding protein 2; (IL): Interleukin; (MMTV): Mouse Mammary tumor Virus; (MOI): Multiplicity of infection; (PON1): Paraoxonase1; (PTHLH): Parathyroid hormone like hormone; (RSPO): R-spondin; (SAA1/2): Serum amyloid proteins; (SUMF1): sulfatase modifying factor 1; (WFDC): WAP Four-Disulfide Core Domain; (wnt): Wingless-type MMTV integration site family

## Competing interests

The authors declare that they have no competing interests.

## Authors' contributions

VHS performed the initial screen, performed the soft agar reconstruction, compiled and analyzed the insert data, cloned library constructs, and drafted the manuscript. TJM participated in discussions and analysis, expanded screen colonies, sequenced colony inserts, cloned library constructs, and helped to draft the manuscript. YCX prepared genomic DNA, performed the sequencing of colony inserts, soft agar staining, and cloned library clones. TYL participated in sequencing, and cloning of library constructs

ZXQ, LKP and OF participated in cloning of library constructs. IL did bioinformatics analysis for the library listing. HWJ conceived of the project, listed the library, participated in discussions, and helped draft the manuscript. All authors have read and approved the final manuscript.

## Supplementary Material

Additional file 1 **The viral vectors pBABE-puro and pBABE-I**. A schematic representation of the vectors used in this study: pBABE-puro and the derived pBABE-I Click here for file

Additional file 2 **Protein library**. List of all proteins in the protein library used in this study. Proteins were selected if they were known to be secreted, contained secretory domains, or bore high homology with secreted proteins. The cloned cDNA's include any signal peptides that may be cleaved off during maturation of the protein. The vast majority of proteins is less than 500 amino acids in length. Click here for file

Additional file 3 **List of DNA inserts from screen colonies**. The DNA inserts recovered from screen colonies are listed, with indication of the protein family they belong to, and their relative recurrence (% of total inserts) Click here for file

Additional file 4 **List of protein families recovered from screen colonies**. The DNA recovered from the screen colonies was grouped in protein families, and the most prevalent protein families are listed here. As multiple protein family members may co-occur in a single colony, the number of colonies may be less than the number of inserts where the protein family is represented. Proteins that have no family members in this screen are listed as individual proteins. Click here for file

Additional file 5 **DNA inserts in each colony**. The actual DNA inserts recovered from each individual colony. Click here for file
