# Supplementary figures and images for: Systematic analysis of secreted proteins reveals synergism between IL6 and other proteins in soft agar growth of MCF10A cells
Source: Cell Biosci. 2011 Mar 25;1:13. doi: 10.1186/2045-3701-1-13 (PMC3125203; doi:10.1186/2045-3701-1-13)

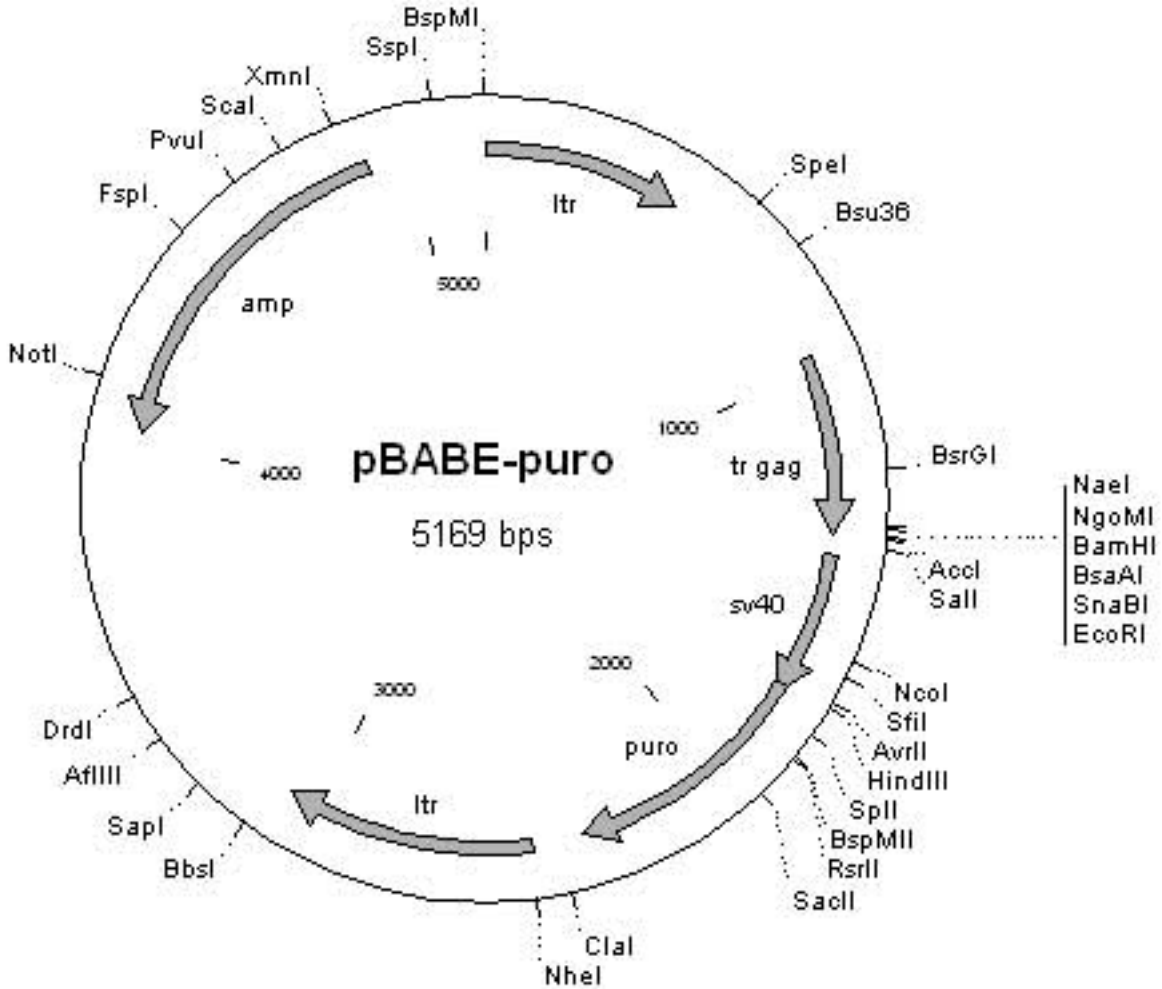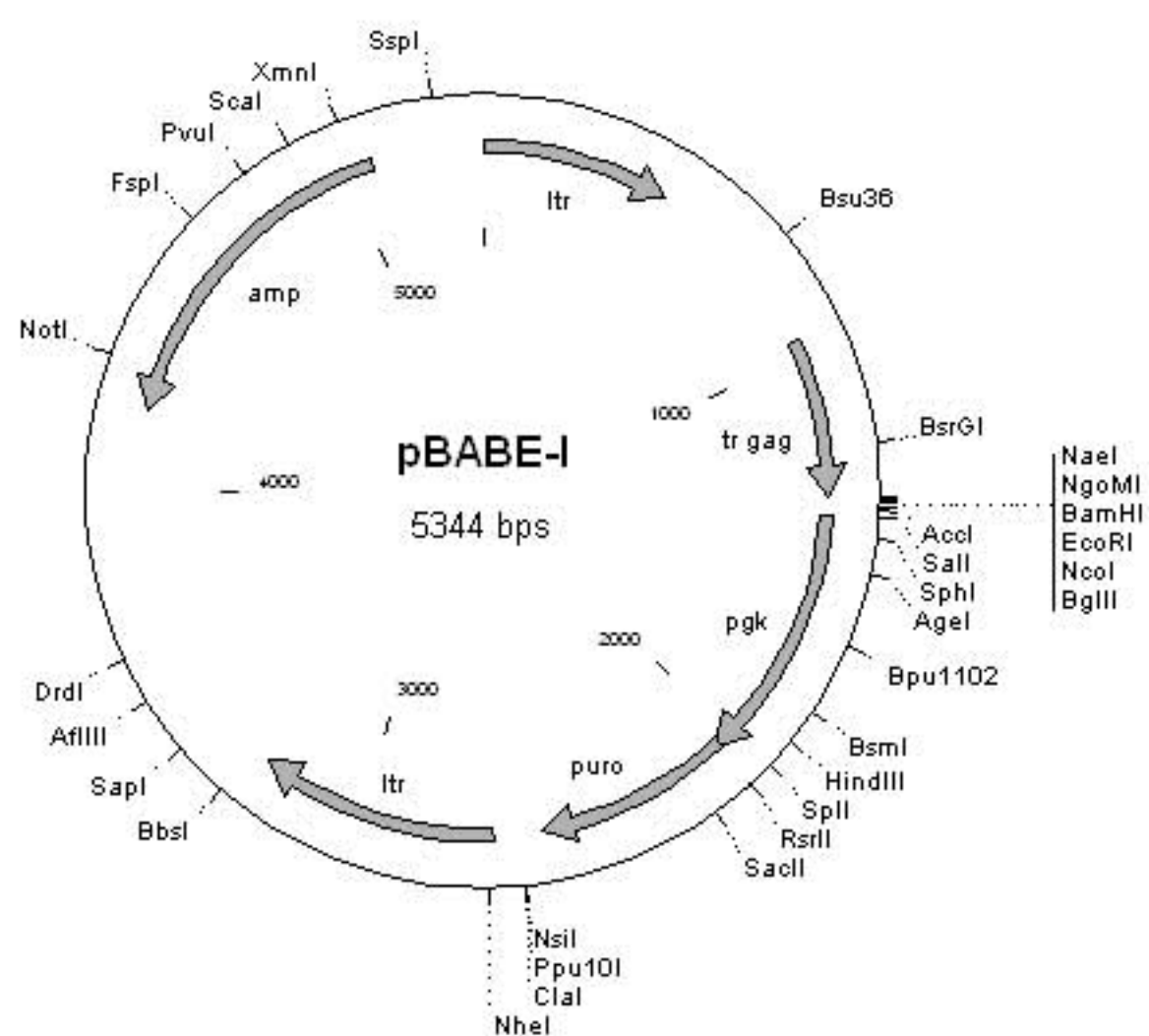

Supplement: Additional file 1 — The viral vectors pBABE-puro and pBABE-I. A schematic representation of the vectors used in this study: pBABE-puro and the derived pBABE-I [file 2045-3701-1-13-S1.PDF]
