# Supplementary material for: Systematic analysis of secreted proteins reveals synergism between IL6 and other proteins in soft agar growth of MCF10A cells
Source: Cell Biosci. 2011 Mar 25;1:13. doi: 10.1186/2045-3701-1-13 (PMC3125203; doi:10.1186/2045-3701-1-13)
Supplement: Additional file 2 — Protein library. List of all proteins in the protein library used in this study. Proteins were selected if they were known to be secreted, contained secretory domains, or bore high homology with secreted proteins. The cloned cDNA's include any signal peptides that may be cleaved off during maturation of the protein. The vast majority of proteins is less than 500 amino acids in length. [file 2045-3701-1-13-S2.DOC]

| Additional table 1: Protein library | | | | |
| --- | --- | --- | --- | --- |
|  | **Designation** | **Protein name** | **Protein ID** | **Amino Acids** |
| 1 | Sec-01-01 | IGFBP1 | NP_000587 | 259 |
| 2 | Sec-01-02 | IGFBP2 | NP_000588 | 328 |
| 3 | Sec-01-03 | IGFBP3 | NP_001013416 | 297 |
| 4 | Sec-01-04 | IGFBP4 | NP_001543 | 258 |
| 5 | Sec-01-05 | IGFBP5 | NP_000590 | 272 |
| 6 | Sec-01-06 | IGFBP6 | NP_002169 | 240 |
| 7 | Sec-01-07 | IGFBP7 | NP_001544 | 282 |
| 8 | Sec-01-08 | CTGF | NP_001892 | 349 |
| 9 | Sec-01-09 | NOV | NP_002505 | 357 |
| 10 | Sec-01-10 | CYR61 | NP_001545 | 381 |
| 11 | Sec-01-11 | WISP1 | NP_003873 | 367 |
| 12 | Sec-01-12 | WISP2 | NP_003872 | 250 |
| 13 | Sec-01-13 | WISP3 | NP_003871 | 354 |
| 14 | Sec-01-14 | WIF1 | NP_009122 | 379 |
| 15 | Sec-01-15 | SOST | NP_079513 | 213 |
| 16 | Sec-01-16 | SOSTDC1 | NP_056279 | 206 |
| 17 | Sec-01-17 | FGFBP1 | NP_005121 | 234 |
| 18 | Sec-01-18 | KSP37 | NP_114156 | 223 |
| 19 | Sec-01-19 | FGFBP3 | NP_689642 | 258 |
| 20 | Sec-01-20 | C1orf56 | NP_060330 | 341 |
| 21 | Sec-02-01 | Sfrp1 | NP_003003 | 314 |
| 22 | Sec-02-02 | Sfrp2 | NP_003004 | 295 |
| 23 | Sec-02-03 | Sfrp3 | NP_001454 | 325 |
| 24 | Sec-02-04 | Sfrp4 | NP_003005 | 346 |
| 25 | Sec-02-05 | Sfrp5 | NP_003006 | 317 |
| 26 | Sec-02-06 | DKK1 | NP_036374 | 266 |
| 27 | Sec-02-07 | DKK2 | NP_055236 | 259 |
| 28 | Sec-02-08 | DKK3 | NP_001018067 | 350 |
| 29 | Sec-02-09 | DKK4 | NP_055235 | 224 |
| 30 | Sec-02-10 | DKKL1 | NP_055234 | 242 |
| 31 | Sec-02-11 | PROK1 | NP_115790 | 105 |
| 32 | Sec-02-12 | PROK2 | NP_068754 | 108 |
| 33 | Sec-02-13 | CRELD1 | NP_001026887 | 422 |
| 34 | Sec-02-14 | CRELD2 | NP_077300 | 353 |
| 35 | Sec-02-15 | RSPO1 | NP_001033722 | 263 |
| 36 | Sec-02-16 | RSPO2 | NP_848660 | 243 |
| 37 | Sec-02-17 | RSPO3 | NP_116173 | 272 |
| 38 | Sec-02-18 | RSPO4 | NP_001025042 | 234 |
| 39 | Sec-02-19 | EGFL7 | NP_057299 | 273 |
| 40 | Sec-02-20 | EGFL8 | NP_085155 | 293 |
| 41 | Sec-03-01 | ANGPT1 | NP_001137 | 498 |
| 42 | Sec-03-03 | ANGPT4 | NP_057069 | 503 |
| 43 | Sec-03-04 | ANGPTL1 | NP_004664 | 491 |
| 44 | Sec-03-06 | ANGPTL3 | NP_055310 | 460 |
| 45 | Sec-03-07 | ANGPTL4 | NP_647475 | 406 |
| 46 | Sec-03-10 | ANGPTL7 | NP_066969 | 346 |
| 47 | Sec-03-11 | FAM3A | NP_068578 | 230 |
| 48 | Sec-03-12 | FAM3B | NP_478066 | 235 |
| 49 | Sec-03-13 | FAM3C | NP_055703 | 227 |
| 50 | Sec-03-14 | FAM3D | NP_620160 | 224 |
| 51 | Sec-03-15 | CRP | NP_000558 | 224 |
| 52 | Sec-03-16 | APCS | NP_001630 | 223 |
| 53 | Sec-03-17 | PTX3 | NP_002843 | 381 |
| 54 | Sec-03-18 | NPTX1 | NP_002513 | 432 |
| 55 | Sec-03-19 | NPTX2 | NP_002514 | 431 |
| 56 | Sec-04-01 | GDF1 | NP_001483 | 372 |
| 57 | Sec-04-02 | GDF2 | NP_057288 | 429 |
| 58 | Sec-04-03 | GDF3 | NP_065685 | 364 |
| 59 | Sec-04-04 | GDF5 | NP_000548 | 501 |
| 60 | Sec-04-05 | GDF6 | NP_001001557 | 455 |
| 61 | Sec-04-07 | GDF8 | NP_005250 | 375 |
| 62 | Sec-04-08 | GDF9 | NP_005251 | 454 |
| 63 | Sec-04-09 | GDF10 | NP_004953 | 478 |
| 64 | Sec-04-10 | GDF11 | NP_005802 | 407 |
| 65 | Sec-04-11 | GDF15 | NP_004855 | 308 |
| 66 | Sec-04-12 | BMP2 | NP_001191 | 396 |
| 67 | Sec-04-13 | BMP3 | NP_001192 | 472 |
| 68 | Sec-04-14 | BMP4 | NP_001193 | 408 |
| 69 | Sec-04-15 | BMP5 | NP_066551 | 454 |
| 70 | Sec-04-16 | BMP6 | NP_001709 | 513 |
| 71 | Sec-04-17 | BMP7 | NP_001710 | 431 |
| 72 | Sec-04-18 | BMP8A | NP_861525 | 402 |
| 73 | Sec-04-19 | BMP10 | NP_055297 | 424 |
| 74 | Sec-04-20 | BMP15 | NP_005439 | 392 |
| 75 | Sec-05-01 | FGF1 | NP_000791 | 155 |
| 76 | Sec-05-03 | FGF3 | NP_005238 | 239 |
| 77 | Sec-05-04 | FGF4 | NP_001998 | 206 |
| 78 | Sec-05-05 | FGF5 | NP_004455 | 268 |
| 79 | Sec-05-06 | FGF6 | NP_066276 | 208 |
| 80 | Sec-05-07 | FGF7 | NP_002000 | 194 |
| 81 | Sec-05-09 | FGF9 | NP_002001 | 208 |
| 82 | Sec-05-10 | FGF10 | NP_004456 | 208 |
| 83 | Sec-05-11 | FGF11 | NP_004103 | 225 |
| 84 | Sec-05-12 | FGF12 | NP_066360 | 243 |
| 85 | Sec-05-13 | FGF13 | NP_004105 | 245 |
| 86 | Sec-05-14 | FGF14 | NP_004106 | 247 |
| 87 | Sec-05-15 | FGF16 | NP_003859 | 207 |
| 88 | Sec-05-16 | FGF17 | NP_003858 | 216 |
| 89 | Sec-05-17 | FGF18 | NP_003853 | 207 |
| 90 | Sec-05-18 | FGF19 | NP_005108 | 216 |
| 91 | Sec-05-19 | FGF20 | NP_062825 | 211 |
| 92 | Sec-05-20 | FGF21 | NP_061986 | 209 |
| 93 | Sec-06-01 | FGF22 | NP_065688 | 177 |
| 94 | Sec-06-02 | FGF23 | NP_065689 | 251 |
| 95 | Sec-06-03 | Wnt1 | NP_005421 | 370 |
| 96 | Sec-06-04 | Wnt2 | NP_003382 | 360 |
| 97 | Sec-06-05 | Wnt2B | NP_078613 | 391 |
| 98 | Sec-06-06 | Wnt3 | NP_110380 | 355 |
| 99 | Sec-06-07 | Wnt4 | NP_110388 | 351 |
| 100 | Sec-06-08 | Wnt5A | NP_003383 | 380 |
| 101 | Sec-06-09 | Wnt6 | NP_006513 | 365 |
| 102 | Sec-06-10 | Wnt7A | NP_004616 | 349 |
| 103 | Sec-06-11 | Wnt8A | NP_490645 | 351 |
| 104 | Sec-06-12 | Wnt8B | NP_003384 | 351 |
| 105 | Sec-06-13 | Wnt9A | NP_003386 | 365 |
| 106 | Sec-06-14 | Wnt9B | NP_003387 | 357 |
| 107 | Sec-06-15 | Wnt10A | NP_079492 | 417 |
| 108 | Sec-06-16 | Wnt10B | NP_003385 | 389 |
| 109 | Sec-06-17 | Wnt11 | NP_004617 | 354 |
| 110 | Sec-06-18 | Wnt16 | NP_476509 | 365 |
| 111 | Sec-06-19 | GKN1 | NP_062563 | 199 |
| 112 | Sec-06-20 | GDDR | NP_872342 | 184 |
| 113 | Sec-07-01 | ADIPOQ | NP_004788 | 244 |
| 114 | Sec-07-02 | C1QA | NP_057075 | 245 |
| 115 | Sec-07-03 | C1QC | NP_758957 | 245 |
| 116 | Sec-07-04 | C1QL1 | NP_006679 | 258 |
| 117 | Sec-07-07 | C1QL4 | NP_001008224 | 238 |
| 118 | Sec-07-08 | C1QTNF1 | NP_112230 | 281 |
| 119 | Sec-07-11 | C1QTNF4 | NP_114115 | 329 |
| 120 | Sec-07-13 | C1QTNF6 | NP_872292 | 259 |
| 121 | Sec-07-14 | C1QTNF7 | NP_114117 | 289 |
| 122 | Sec-07-17 | CBLN2 | NP_872317 | 224 |
| 123 | Sec-07-18 | CBLN3 | NP_001034860 | 205 |
| 124 | Sec-07-19 | CBLN4 | NP_542184 | 201 |
| 125 | Sec-07-20 | LOC348174 | NP_872425 | 446 |
| 126 | Sec-08-01 | REG1A | NP_002900 | 166 |
| 127 | Sec-08-02 | REG3A | NP_620355 | 175 |
| 128 | Sec-08-03 | REG3G | NP_940850 | 175 |
| 129 | Sec-08-04 | REG4 | NP_114433 | 158 |
| 130 | Sec-08-05 | COLEC10 | NP_006429 | 277 |
| 131 | Sec-08-07 | CLEC3A | NP_005743 | 197 |
| 132 | Sec-08-08 | CLEC3B | NP_003269 | 202 |
| 133 | Sec-08-09 | CLEC11A | NP_002966 | 323 |
| 134 | Sec-08-10 | PRG1 | NP_002718 | 158 |
| 135 | Sec-08-11 | PRG2 | NP_002719 | 222 |
| 136 | Sec-08-12 | PRG3 | NP_006084 | 225 |
| 137 | Sec-08-13 | MBL2 | NP_000233 | 248 |
| 138 | Sec-08-14 | FCN1 | NP_001994 | 326 |
| 139 | Sec-08-15 | FCN2 | NP_004099 | 313 |
| 140 | Sec-08-16 | FCN3 | NP_003656 | 299 |
| 141 | Sec-08-17 | MASP1 | NP_001027019 | 380 |
| 142 | Sec-08-18 | MASP2 | NP_631947 | 185 |
| 143 | Sec-08-19 | SFTPA1 | NP_005402 | 248 |
| 144 | Sec-08-20 | SFTPB | NP_942140 | 381 |
| 145 | Sec-09-01 | SFTPC | NP_003009 | 197 |
| 146 | Sec-09-02 | SFTPD | NP_003010 | 375 |
| 147 | Sec-09-03 | IL4 | NP_000580 | 153 |
| 148 | Sec-09-04 | IL7 | NP_000871 | 177 |
| 149 | Sec-09-05 | IL9 | NP_000581 | 144 |
| 150 | Sec-09-06 | IL15 | NP_000576 | 162 |
| 151 | Sec-09-07 | IL21 | NP_068575 | 162 |
| 152 | Sec-09-08 | IL17 | NP_002181 | 155 |
| 153 | Sec-09-09 | IL17B | NP_055258 | 180 |
| 154 | Sec-09-10 | IL17C | NP_037410 | 197 |
| 155 | Sec-09-11 | IL17D | NP_612141 | 202 |
| 156 | Sec-09-12 | IL17E | NP_073626 | 177 |
| 157 | Sec-09-13 | IL17F | NP_443104 | 163 |
| 158 | Sec-09-14 | IL8 | NP_000575 | 99 |
| 159 | Sec-09-15 | IL25 | NP_061980 | 173 |
| 160 | Sec-09-17 | IL12B | NP_002178 | 328 |
| 161 | Sec-09-18 | EBI3 | NP_005746 | 229 |
| 162 | Sec-09-19 | PTN | NP_002816 | 168 |
| 163 | Sec-09-20 | MDK | NP_002382 | 143 |
| 164 | Sec-10-01 | IL2 | NP_000577 | 153 |
| 165 | Sec-10-02 | IL3 | NP_000579 | 152 |
| 166 | Sec-10-03 | IL5 | NP_000870 | 134 |
| 167 | Sec-10-04 | IL6 | NP_000591 | 212 |
| 168 | Sec-10-05 | IL10 | NP_000563 | 178 |
| 169 | Sec-10-06 | IL11 | NP_000632 | 199 |
| 170 | Sec-10-07 | IL12A | NP_000873 | 253 |
| 171 | Sec-10-08 | IL13 | NP_002179 | 146 |
| 172 | Sec-10-09 | IL19 | NP_715639 | 215 |
| 173 | Sec-10-10 | IL20 | NP_061194 | 176 |
| 174 | Sec-10-11 | IL22 | NP_065386 | 179 |
| 175 | Sec-10-12 | IL24 | NP_006841 | 206 |
| 176 | Sec-10-13 | IL26 | NP_060872 | 171 |
| 177 | Sec-10-14 | IL27 | NP_663634 | 248 |
| 178 | Sec-10-15 | IL28A | NP_742150 | 200 |
| 179 | Sec-10-16 | IL29 | NP_742152 | 200 |
| 180 | Sec-10-17 | IL31 | NP_001014358 | 164 |
| 181 | Sec-10-18 | AGR2 | NP_006399 | 175 |
| 182 | Sec-10-19 | BCMP11 | NP_789783 | 166 |
| 183 | Sec-10-20 | TLP19 | NP_056997 | 172 |
| 184 | Sec-11-01 | WFDC1 | NP_067020 | 220 |
| 185 | Sec-11-02 | WFDC2 | NP_006094 | 124 |
| 186 | Sec-11-04 | WFDC4 | NP_003055 | 132 |
| 187 | Sec-11-05 | WFDC5 | NP_663627 | 123 |
| 188 | Sec-11-06 | WFDC7 | NP_065131 | 133 |
| 189 | Sec-11-07 | WFDC8 | NP_570966 | 241 |
| 190 | Sec-11-09 | WFDC11 | NP_671730 | 87 |
| 191 | Sec-11-11 | WFDC13 | NP_742002 | 93 |
| 192 | Sec-11-12 | WFDC14 | NP_002629 | 117 |
| 193 | Sec-11-13 | IFNA1 | NP_076918 | 189 |
| 194 | Sec-11-15 | IFNE1 | NP_795372 | 208 |
| 195 | Sec-11-16 | IFNG | NP_000610 | 166 |
| 196 | Sec-11-18 | IFNW1 | NP_002168 | 195 |
| 197 | Sec-11-19 | CHRDL1 | NP_660277 | 456 |
| 198 | Sec-11-20 | CHRDL2 | NP_056239 | 451 |
| 199 | Sec-12-01 | CXCL1 | NP_001502 | 107 |
| 200 | Sec-12-02 | CXCL2 | NP_002080 | 107 |
| 201 | Sec-12-03 | CXCL3 | NP_002081 | 107 |
| 202 | Sec-12-05 | CXCL5 | NP_002985 | 114 |
| 203 | Sec-12-06 | CXCL6 | NP_002984 | 114 |
| 204 | Sec-12-07 | CXCL7 | NP_002695 | 128 |
| 205 | Sec-12-08 | CXCL8 | NP_000575 | 99 |
| 206 | Sec-12-09 | CXCL9 | NP_002407 | 125 |
| 207 | Sec-12-10 | CXCL10 | NP_001556 | 98 |
| 208 | Sec-12-11 | CXCL11 | NP_005400 | 94 |
| 209 | Sec-12-12 | CXCL12 | NP_000600 | 93 |
| 210 | Sec-12-13 | CXCL13 | NP_006410 | 109 |
| 211 | Sec-12-14 | CXCL14 | NP_004878 | 111 |
| 212 | Sec-12-15 | CXCL16 | NP_071342 | 254 |
| 213 | Sec-12-16 | CCL1 | NP_002972 | 96 |
| 214 | Sec-12-17 | CCL2 | NP_002973 | 99 |
| 215 | Sec-12-18 | CCL3 | NP_002974 | 92 |
| 216 | Sec-12-19 | CCL3L1 | NP_066286 | 93 |
| 217 | Sec-12-20 | CCL4 | NP_002975 | 92 |
| 218 | Sec-13-01 | CCL4L2 | NP_996890 | 92 |
| 219 | Sec-13-02 | CCL5 | NP_002976 | 91 |
| 220 | Sec-13-03 | CCL7 | NP_006264 | 99 |
| 221 | Sec-13-04 | CCL8 | NP_005614 | 99 |
| 222 | Sec-13-05 | CCL11 | NP_002977 | 97 |
| 223 | Sec-13-06 | CCL13 | NP_005399 | 98 |
| 224 | Sec-13-07 | CCL14 | NP_004157 | 93 |
| 225 | Sec-13-08 | CCL15 | NP_116741 | 113 |
| 226 | Sec-13-09 | CCL16 | NP_004581 | 120 |
| 227 | Sec-13-10 | CCL17 | NP_002978 | 94 |
| 228 | Sec-13-11 | CCL18 | NP_002979 | 89 |
| 229 | Sec-13-12 | CCL19 | NP_006265 | 98 |
| 230 | Sec-13-13 | CCL20 | NP_004582 | 96 |
| 231 | Sec-13-14 | CCL21 | NP_002980 | 134 |
| 232 | Sec-13-15 | CCL22 | NP_002981 | 93 |
| 233 | Sec-13-16 | CCL23 | NP_665905 | 120 |
| 234 | Sec-13-17 | CCL24 | NP_002982 | 119 |
| 235 | Sec-13-18 | CCL25 | NP_005615 | 150 |
| 236 | Sec-13-19 | CCL26 | NP_006063 | 94 |
| 237 | Sec-13-20 | CCL27 | NP_006655 | 112 |
| 238 | Sec-14-01 | CCL28 | NP_683513 | 127 |
| 239 | Sec-14-02 | SPAG11 | NP_057596 | 103 |
| 240 | Sec-14-03 | DEFA1 | NP_004075 | 94 |
| 241 | Sec-14-04 | DEFA4 | NP_001916 | 97 |
| 242 | Sec-14-05 | DEFA5 | NP_066290 | 94 |
| 243 | Sec-14-06 | DEFA6 | NP_001917 | 100 |
| 244 | Sec-14-07 | DEFA108B | NP_001002035 | 73 |
| 245 | Sec-14-08 | DEFB4 | NP_004933 | 64 |
| 246 | Sec-14-09 | DEFB32 | NP_997352 | 95 |
| 247 | Sec-14-10 | DEFB103A | NP_061131 | 67 |
| 248 | Sec-14-11 | DEFB104A | NP_525128 | 72 |
| 249 | Sec-14-12 | DEFB105A | NP_689463 | 78 |
| 250 | Sec-14-13 | DEFB106A | NP_689464 | 65 |
| 251 | Sec-14-15 | DEFB110 | NP_001032817 | 62 |
| 252 | Sec-14-16 | DEFB111 | NP_001032586 | 67 |
| 253 | Sec-14-17 | DEFB112 | NP_001032587 | 113 |
| 254 | Sec-14-18 | DEFB113 | NP_001032818 | 82 |
| 255 | Sec-14-19 | DEFB114 | NP_001032588 | 69 |
| 256 | Sec-15-02 | DEFB118 | NP_473453 | 123 |
| 257 | Sec-15-03 | DEFB119 | NP_695021 | 84 |
| 258 | Sec-15-04 | DEFB121 | NP_001011878 | 76 |
| 259 | Sec-15-05 | DEFB123 | NP_697019 | 67 |
| 260 | Sec-15-06 | DEFB124 | NP_001032589 | 71 |
| 261 | Sec-15-07 | DEFB125 | NP_697020 | 156 |
| 262 | Sec-15-08 | DEFB126 | NP_112193 | 111 |
| 263 | Sec-15-09 | DEFB127 | NP_620713 | 99 |
| 264 | Sec-15-10 | DEFB128 | NP_001032821 | 93 |
| 265 | Sec-15-11 | DEFB129 | NP_543021 | 183 |
| 266 | Sec-15-12 | DEFB130 | NP_001032893 | 79 |
| 267 | Sec-15-13 | LBP | NP_004130 | 481 |
| 268 | Sec-15-14 | BPI | NP_001716 | 487 |
| 269 | Sec-15-15 | PLTP | NP_006218 | 493 |
| 270 | Sec-15-16 | BPIL1 | NP_079503 | 458 |
| 271 | Sec-15-17 | BPIL2 | NP_777592 | 507 |
| 272 | Sec-15-18 | BPIL3 | NP_777557 | 453 |
| 273 | Sec-15-19 | CETP | NP_000069 | 493 |
| 274 | Sec-15-20 | SCRG1 | NP_009212 | 98 |
| 275 | Sec-16-01 | HAMP | NP_066998 | 84 |
| 276 | Sec-16-02 | UNQ467 | NP_997275 | 99 |
| 277 | Sec-16-03 | RETN | NP_065148 | 108 |
| 278 | Sec-16-04 | RETNLB | NP_115968 | 111 |
| 279 | Sec-16-06 | IHH | NP_002172 | 411 |
| 280 | Sec-16-08 | BDNF | NP_001700 | 247 |
| 281 | Sec-16-09 | NGFB | NP_002497 | 241 |
| 282 | Sec-16-10 | NTF3 | NP_002518 | 257 |
| 283 | Sec-16-11 | NTF5 | NP_006170 | 210 |
| 284 | Sec-16-12 | TGFB1 | NP_000651 | 390 |
| 285 | Sec-16-13 | TGFB2 | NP_003229 | 414 |
| 286 | Sec-16-14 | TGFB3 | NP_003230 | 412 |
| 287 | Sec-16-15 | LEFTY2 | NP_003231 | 366 |
| 288 | Sec-16-16 | INHA | NP_002182 | 366 |
| 289 | Sec-16-17 | INHBA | NP_002183 | 426 |
| 290 | Sec-16-20 | INHBE | NP_113667 | 350 |
| 291 | Sec-17-01 | FST | NP_037541 | 344 |
| 292 | Sec-17-02 | FSTL1 | NP_009016 | 308 |
| 293 | Sec-17-03 | FSTL3 | NP_005851 | 263 |
| 294 | Sec-17-04 | WFIKKN1 | NP_444514 | 548 |
| 295 | Sec-17-05 | WFIKKN2 | NP_783165 | 576 |
| 296 | Sec-17-06 | PGLYRP1 | NP_005082 | 196 |
| 297 | Sec-17-08 | PGLYRP3 | NP_443123 | 341 |
| 298 | Sec-17-10 | LY86 | NP_004262 | 162 |
| 299 | Sec-17-11 | LY96 | NP_056179 | 160 |
| 300 | Sec-17-12 | GM2A | NP_000396 | 193 |
| 301 | Sec-17-13 | NPC2 | NP_006423 | 151 |
| 302 | Sec-17-14 | TIMP1 | NP_003245 | 207 |
| 303 | Sec-17-15 | TIMP2 | NP_003246 | 220 |
| 304 | Sec-17-16 | TIMP3 | NP_000353 | 211 |
| 305 | Sec-17-17 | TIMP4 | NP_003247 | 224 |
| 306 | Sec-17-19 | QSCN6L1 | NP_859052 | 698 |
| 307 | Sec-17-20 | UTS2 | NP_068835 | 139 |
| 308 | Sec-18-01 | TEX264 | NP_057010 | 313 |
| 309 | Sec-18-03 | AZU1 | NP_001691 | 251 |
| 310 | Sec-18-04 | PRSSL1 | NP_999875 | 283 |
| 311 | Sec-18-05 | ELA2 | NP_001963 | 267 |
| 312 | Sec-18-06 | PRTN3 | NP_002768 | 256 |
| 313 | Sec-18-07 | CAMP | NP_004336 | 170 |
| 314 | Sec-18-08 | RNASE3 | NP_002926 | 160 |
| 315 | Sec-18-10 | CRISP2 | NP_003287 | 243 |
| 316 | Sec-18-11 | CRISP3 | NP_006052 | 245 |
| 317 | Sec-18-14 | PI15 | NP_056970 | 258 |
| 318 | Sec-18-17 | LYNX1 | NP_076435 | 131 |
| 319 | Sec-18-18 | LYPD1 | NP_653187 | 165 |
| 320 | Sec-18-19 | TNFAIP6 | NP_009046 | 277 |
| 321 | Sec-18-20 | GRN | NP_002078 | 593 |
| 322 | Sec-19-01 | CST1 | NP_001889 | 141 |
| 323 | Sec-19-02 | CST2 | NP_001313 | 141 |
| 324 | Sec-19-03 | CST3 | NP_000090 | 146 |
| 325 | Sec-19-04 | CST4 | NP_001890 | 141 |
| 326 | Sec-19-05 | CST5 | NP_001891 | 142 |
| 327 | Sec-19-06 | CST6 | NP_001314 | 149 |
| 328 | Sec-19-07 | CST7 | NP_003641 | 167 |
| 329 | Sec-19-08 | CST8 | NP_005483 | 142 |
| 330 | Sec-19-09 | CST9 | NP_001008693 | 159 |
| 331 | Sec-19-10 | CST9L | NP_542177 | 147 |
| 332 | Sec-19-11 | CST11 | NP_570612 | 138 |
| 333 | Sec-19-12 | CSTL1 | NP_612140 | 145 |
| 334 | Sec-19-13 | SERPINF1 | NP_002606 | 416 |
| 335 | Sec-19-14 | SERPINF2 | NP_000925 | 491 |
| 336 | Sec-19-15 | SERPINA1 | NP_001002236 | 418 |
| 337 | Sec-19-16 | SERPINC1 | NP_000479 | 464 |
| 338 | Sec-19-17 | AHSG | NP_001613 | 367 |
| 339 | Sec-19-18 | OIT3 | NP_689848 | 545 |
| 340 | Sec-19-19 | HABP2 | NP_004123 | 560 |
| 341 | Sec-19-20 | VTN | NP_000629 | 478 |
| 342 | Sec-20-01 | KLK1 | NP_002248 | 262 |
| 343 | Sec-20-02 | KLK2 | NP_005542 | 261 |
| 344 | Sec-20-03 | KLK3 | NP_001639 | 261 |
| 345 | Sec-20-04 | KLK4 | NP_004908 | 254 |
| 346 | Sec-20-05 | KLK5 | NP_036559 | 293 |
| 347 | Sec-20-06 | KLK6 | NP_002765 | 244 |
| 348 | Sec-20-07 | KLK7 | NP_644806 | 253 |
| 349 | Sec-20-08 | KLK8 | NP_009127 | 260 |
| 350 | Sec-20-09 | KLK9 | NP_036447 | 250 |
| 351 | Sec-20-10 | KLK10 | NP_665895 | 276 |
| 352 | Sec-20-11 | KLK11 | NP_006844 | 250 |
| 353 | Sec-20-12 | KLK12 | NP_062544 | 254 |
| 354 | Sec-20-13 | KLK13 | NP_056411 | 277 |
| 355 | Sec-20-14 | KLK14 | NP_071329 | 267 |
| 356 | Sec-20-15 | KLK15 | NP_059979 | 256 |
| 357 | Sec-20-16 | CFHR4 | NP_006675 | 331 |
| 358 | Sec-20-17 | RBP4 | NP_006735 | 201 |
| 359 | Sec-20-18 | LEAP2 | NP_443203 | 77 |
| 360 | Sec-21-01 | SPP2 | NP_008875 | 211 |
| 361 | Sec-21-03 | SAA4 | NP_006503 | 130 |
| 362 | Sec-21-04 | IGFALS | NP_004961 | 605 |
| 363 | Sec-21-05 | LECT2 | NP_002293 | 151 |
| 364 | Sec-21-06 | LCAT | NP_000220 | 440 |
| 365 | Sec-21-07 | A1BG | NP_570602 | 495 |
| 366 | Sec-21-08 | PRAP1 | NP_660203 | 151 |
| 367 | Sec-21-09 | PON1 | NP_000437 | 355 |
| 368 | Sec-21-10 | PON2 | NP_000296 | 354 |
| 369 | Sec-21-11 | PON3 | NP_000931 | 354 |
| 370 | Sec-21-12 | AMBP | NP_001624 | 352 |
| 371 | Sec-21-13 | SMOC1 | NP_071420 | 434 |
| 372 | Sec-21-15 | CHAD | NP_001258 | 359 |
| 373 | Sec-21-16 | CSF3 | NP_000750 | 207 |
| 374 | Sec-21-17 | EGFL6 | NP_056322 | 553 |
| 375 | Sec-21-18 | ESM1 | NP_008967 | 184 |
| 376 | Sec-21-19 | STC1 | NP_003146 | 247 |
| 377 | Sec-21-20 | STC2 | NP_003705 | 302 |
| 378 | Sec-22-04 | FBLN1 | NP_001987 | 683 |
| 379 | Sec-22-05 | F10 | NP_000495 | 488 |
| 380 | Sec-22-09 | LIF | NP_002300 | 202 |
| 381 | Sec-22-10 | MIA | NP_006524 | 131 |
| 382 | Sec-22-11 | OTOR | NP_064542 | 128 |
| 383 | Sec-22-16 | SCGB3A1 | NP_443095 | 104 |
| 384 | Sec-22-17 | SPARC | NP_003109 | 303 |
| 385 | Sec-22-18 | SPARCL1 | NP_004675 | 664 |
| 386 | Sec-22-19 | SPON1 | NP_006099 | 807 |
| 387 | Sec-22-20 | SPON2 | NP_036577 | 331 |
| 388 | Sec-23-01 | SPP1 | NP_000573 | 300 |
| 389 | Sec-23-02 | TGFBI | NP_000349 | 683 |
| 390 | Sec-23-03 | THPO | NP_000451 | 353 |
| 391 | Sec-23-04 | DCN | NP_598010 | 359 |
| 392 | Sec-23-05 | ASPN | NP_060150 | 380 |
| 393 | Sec-23-06 | BGN | NP_001702 | 368 |
| 394 | Sec-23-08 | FGB | NP_005132 | 491 |
| 395 | Sec-23-09 | CHI3L1 | NP_001267 | 383 |
| 396 | Sec-23-10 | CHI3L2 | NP_003991 | 390 |
| 397 | Sec-23-11 | CHIT1 | NP_003456 | 466 |
| 398 | Sec-23-12 | TNFRSF11B | NP_002537 | 401 |
| 399 | Sec-23-13 | CLU | NP_001822 | 448 |
| 400 | Sec-23-14 | THBS1 | NP_003237 | 1170 |
| 401 | Sec-23-15 | THBS2 | NP_003238 | 1172 |
| 402 | Sec-23-16 | THBS3 | NP_009043 | 956 |
| 403 | Sec-23-17 | THBS4 | NP_003239 | 961 |
| 404 | Sec-23-18 | SCGB1D1 | NP_006543 | 90 |
| 405 | Sec-23-19 | SCGB1D2 | NP_006542 | 90 |
| 406 | Sec-23-20 | SCGB1D4 | NP_996881 | 83 |
| 407 | Sec-24-01 | A1BG | NP_570602 | 495 |
| 408 | Sec-24-03 | APOD | NP_001638 | 189 |
| 409 | Sec-24-04 | APOH | NP_000033 | 345 |
| 410 | Sec-24-05 | AZGP1 | NP_001176 | 298 |
| 411 | Sec-24-06 | CFB | NP_001701 | 764 |
| 412 | Sec-24-08 | CLEC2B | NP_005118 | 149 |
| 413 | Sec-24-09 | CLEC5A | NP_037384 | 188 |
| 414 | Sec-24-10 | COMP | NP_000086 | 757 |
| 415 | Sec-24-12 | PROZ | NP_003882 | 400 |
| 416 | Sec-24-15 | F10 | NP_000495 | 488 |
| 417 | Sec-24-17 | GIF | NP_005133 | 417 |
| 418 | Sec-24-18 | TCN1 | NP_001053 | 433 |
| 419 | Sec-24-19 | PPGB | NP_000299 | 480 |
| 420 | Sec-25-01 | LUM | NP_002336 | 338 |
| 421 | Sec-25-02 | KERA | NP_008966 | 352 |
| 422 | Sec-25-03 | OMD | NP_005005 | 421 |
| 423 | Sec-25-08 | LCN1 | NP_002288 | 186 |
| 424 | Sec-25-09 | PTGDS | NP_000945 | 190 |
| 425 | Sec-25-12 | PRB3 | NP_006240 | 351 |
| 426 | Sec-25-13 | PRB4 | NP_002714 | 247 |
| 427 | Sec-25-16 | NBL1 | NP_005371 | 180 |
| 428 | Sec-25-17 | GREM1 | NP_037504 | 184 |
| 429 | Sec-25-18 | GREM2 | NP_071914 | 168 |
| 430 | Sec-25-19 | DAND5 | NP_689867 | 189 |
| 431 | Sec-26-01 | KDELC1 | NP_076994 | 502 |
| 432 | Sec-26-02 | KDELC2 | NP_714916 | 507 |
| 433 | Sec-26-03 | C3orf9 | NP_064616 | 392 |
| 434 | Sec-26-05 | RCN2 | NP_002893 | 317 |
| 435 | Sec-26-06 | RCN3 | NP_065701 | 328 |
| 436 | Sec-26-07 | CALU | NP_001210 | 315 |
| 437 | Sec-26-08 | SDF4 | NP_057631 | 348 |
| 438 | Sec-26-09 | SERPINA3 | NP_001076 | 423 |
| 439 | Sec-26-10 | SAA1 | NP_000322 | 122 |
| 440 | Sec-26-11 | ORM1 | NP_000598 | 201 |
| 441 | Sec-26-12 | EBAG9 | NP_936056 | 213 |
| 442 | Sec-26-14 | TPP1 | NP_000382 | 563 |
| 443 | Sec-26-15 | SUMF1 | NP_877437 | 374 |
| 444 | Sec-26-17 | IFI30 | NP_006323 | 250 |
| 445 | Sec-26-19 | P4HA2 | NP_004190 | 535 |
| 446 | Sec-26-20 | P4HA3 | NP_878907 | 544 |
| 447 | Sec-27-01 | CHGA | NP_001266 | 457 |
| 448 | Sec-27-02 | CHGB | NP_001810 | 677 |
| 449 | Sec-27-03 | SCG2 | NP_003460 | 617 |
| 450 | Sec-27-04 | SCG3 | NP_037375 | 468 |
| 451 | Sec-27-05 | SCG5 | NP_003011 | 211 |
| 452 | Sec-27-06 | SPOCK1 | NP_004589 | 439 |
| 453 | Sec-27-07 | ECM1 | NP_004416 | 540 |
| 454 | Sec-27-09 | NODAL | NP_060525 | 347 |
| 455 | Sec-27-10 | ZG16 | NP_689551 | 167 |
| 456 | Sec-27-11 | PTHLH | NP_945317 | 177 |
| 457 | Sec-27-16 | PLA1A | NP_056984 | 456 |
| 458 | Sec-27-17 | LPL | NP_000228 | 475 |
| 459 | Sec-28-03 | OGN | NP_077727 | 298 |
| 460 | Sec-28-04 | FMOD | NP_002014 | 376 |
| 461 | Sec-28-05 | BMPER | NP_597725 | 685 |
| 462 | Sec-28-15 | SRPX | NP_006298 | 464 |
| 463 | Sec-28-16 | SRPX2 | NP_055282 | 465 |
| 464 | Sec-28-18 | MGP | NP_000891 | 103 |
| 465 | Sec-28-19 | EDIL1 | NP_005919 | 387 |
| 466 | Sec-28-20 | EDIL3 | NP_005702 | 480 |
| 467 | Sec-34-15 | IL23A | NP_057668 | 189 |
