# Supplementary material for: Systematic analysis of secreted proteins reveals synergism between IL6 and other proteins in soft agar growth of MCF10A cells
Source: Cell Biosci. 2011 Mar 25;1:13. doi: 10.1186/2045-3701-1-13 (PMC3125203; doi:10.1186/2045-3701-1-13)
Supplement: Additional file 3 — List of DNA inserts from screen colonies. The DNA inserts recovered from screen colonies are listed, with indication of the protein family they belong to, and their relative recurrence (% of total inserts) [file 2045-3701-1-13-S3.DOC]

| **Additional table 2: List of DNA inserts from screen colonies** | | | | |
| --- | --- | --- | --- | --- |
| **total inserts** | **% of all inserts** | **protein** | **Protein family** | **Description** |
| **68** | 6.9% | **WFDC13** | WFDC | WAP four-disulfide core domain 13 |
| **36** | 3.6% | **PON1** | PON | paraoxonase 1 |
| **31** | 3.1% | **Wnt3** | Wnt | wingless-type MMTV integration site family, member 3 |
| **18** | 1.8% | **FGFBP2** | FGFBP | fibroblast growth factor binding protein 2 |
| **15** | 1.5% | **IL6** | Interleukin | interleukin 6 (interferon, beta 2) |
| **15** | 1.5% | **FGF14** | FGF | fibroblast growth factor 14 |
| **14** | 1.4% | **SAA1** | SAA | serum amyloid A1 |
| **14** | 1.4% | **PTHLH** | PTHLH | parathyroid hormone-like hormone |
| **13** | 1.3% | **FGF10** | FGF | fibroblast growth factor 10 |
| **12** | 1.2% | **LYNX1** | Ly-6 | Ly6/neurotoxin 1 |
| **12** | 1.2% | **IL21** | Interleukin | interleukin 21 |
| **11** | 1.1% | **IL22** | Interleukin | interleukin 22 |
| **10** | 1.0% | **WFDC14** | WFDC | peptidase inhibitor 3, skin-derived (SKALP) |
| **10** | 1.0% | **SUMF1** | SUMF | sulfatase modifying factor 1 |
| **10** | 1.0% | **FGF1** | FGF | fibroblast growth factor 1 (acidic) |
| **9** | 0.9% | **NODAL** | TGFB | nodal homolog (mouse) |
| **9** | 0.9% | **IL27** | Interleukin | interleukin 27 |
| **9** | 0.9% | **AZU1** | AZU | azurocidin 1 (cationic antimicrobial protein 37) |
| **8** | 0.8% | **TIMP3** | TIMP | TIMP metallopeptidase inhibitor 3 |
| **8** | 0.8% | **RSPO3** | RSPO | R-spondin 3 homolog (Xenopus laevis) |
| **8** | 0.8% | **MASP2** | MASP | mannan-binding lectin serine peptidase 2 |
| **8** | 0.8% | **GDF8** | TGFB | myostatin |
| **8** | 0.8% | **APOD** | APO | apolipoprotein D |
| **7** | 0.7% | **LY86** | Ly86 | lymphocyte antigen 86 |
| **7** | 0.7% | **WFDC4/SLPI** | WFDC | secretory leukocyte peptidase inhibitor |
| **7** | 0.7% | **UTS2** | UTS | urotensin 2 |
| **7** | 0.7% | **TIMP4** | TIMP | TIMP metallopeptidase inhibitor 4 |
| **7** | 0.7% | **PGLYRP1** | PGLYRP | peptidoglycan recognition protein 1 |
| **7** | 0.7% | **TIMP1** | TIMP | TIMP metallopeptidase inhibitor 1 |
| **6** | 0.6% | **Wnt9a** | Wnt | wingless-type MMTV integration site family, member 9A |
| **6** | 0.6% | **WFDC11** | WFDC | WAP four-disulfide core domain 11 |
| **6** | 0.6% | **SMOC1** | SPARC | SPARC related modular calcium binding 1 |
| **6** | 0.6% | **SFTPA2** | SFTP | surfactant, pulmonary-associated protein A2 |
| **6** | 0.6% | **PGLYRP3** | PGLYRP | peptidoglycan recognition protein 3 |
| **6** | 0.6% | **ORM1** | ORM | orosomucoid 1 |
| **6** | 0.6% | **LPL** | lipase | lipoprotein lipase |
| **6** | 0.6% | **KLK2** | KLK | kallikrein-related peptidase 3 |
| **6** | 0.6% | **KLK3** | KLK | kallikrein-related peptidase 2 |
| **6** | 0.6% | **IL9** | Interleukin | interleukin 9 |
| **6** | 0.6% | **IGFBP5** | IGFBP | insulin-like growth factor binding protein 5 |
| **6** | 0.6% | **IFNA1** | IFN | interferon, alpha 1 |
| **6** | 0.6% | **FGF23** | FGF | fibroblast growth factor 23 |
| **6** | 0.6% | **DKK1** | DKK | dickkopf homolog 1 (Xenopus laevis) |
| **5** | 0.5% | **Wnt8A** | WNT | wingless-type MMTV integration site family, member 8A |
| **5** | 0.5% | **Wnt2** | Wnt | wingless-type MMTV integration site family member 2 |
| **5** | 0.5% | **WISP3** | WISP | WNT1 inducible signaling pathway protein 3 |
| **5** | 0.5% | **SPON2** | RSPO | spondin 2, extracellular matrix protein |
| **5** | 0.5% | **SFTPC** | SFTP | surfactant, pulmonary-associated protein C |
| **5** | 0.5% | **KLK15** | KLK | kallikrein-related peptidase 15 |
| **5** | 0.5% | **IL31** | Interleukin | interleukin 31 |
| **5** | 0.5% | **IL20** | Interleukin | interleukin 20 |
| **5** | 0.5% | **IL12B** | Interleukin | interleukin |
| **5** | 0.5% | **FGF4** | FGF | fibroblast growth factor 4 |
| **5** | 0.5% | **FAM3B** | FAM | family with sequence similarity 3, member B |
| **5** | 0.5% | **DEFB130** | DEF | defensin, beta 130 |
| **5** | 0.5% | **C1QTNF6** | C1Q | C1q and tumor necrosis factor related protein 6 |
| **5** | 0.5% | **APOH** | APO | apolipoprotein H (beta-2-glycoprotein I) |
| **4** | 0.4% | **WISP1** | WISP | WNT1 inducible signaling pathway protein 1 |
| **4** | 0.4% | **WFDC7** | WFDC | serine peptidase inhibitor-like, with Kunitz and WAP domains 1 |
| **4** | 0.4% | **TIMP2** | TIMP | TIMP metallopeptidase inhibitor 2 |
| **4** | 0.4% | **TEX264** | TEX | testis expressed 264 |
| **4** | 0.4% | **STC2** | STC | stanniocalcin 2 |
| **4** | 0.4% | **STC1** | STC | stanniocalcin 1 |
| **4** | 0.4% | **SRPX** | SRPX | sushi-repeat-containing protein, X-linked |
| **4** | 0.4% | **RCN2** | RCN | reticulocalbin 2, EF-hand calcium binding domain |
| **4** | 0.4% | **PLA1A** | PLA1A | phospholipase A1 member A |
| **4** | 0.4% | **PI15** | PI | peptidase inhibitor 15 |
| **4** | 0.4% | **MDK** | Interleukin | midkine (neurite growth-promoting factor 2) |
| **4** | 0.4% | **MASP1** | MASP | mannan-binding lectin serine peptidase 1 |
| **4** | 0.4% | **KLK6** | KLK | kallikrein-related peptidase 6 |
| **4** | 0.4% | **KLK14** | KLK | kallikrein-related peptidase 14 |
| **4** | 0.4% | **IL17** | Interleukin | interleukin 17A |
| **4** | 0.4% | **IGFALS** | IGFBP | insulin-like growth factor binding protein, acid labile subunit |
| **4** | 0.4% | **FMOD** | FMOD | fibromodulin |
| **4** | 0.4% | **FGF9** | FGF | fibroblast growth factor 9 (glia-activating factor) |
| **4** | 0.4% | **FGF3** | FGF | fibroblast growth factor 3 |
| **4** | 0.4% | **DKK2** | DKK | dickkopf homolog 2 (Xenopus laevis) |
| **4** | 0.4% | **CXCL7** | CXCL | pro-platelet basic protein (chemokine (C-X-C motif) ligand 7) |
| **4** | 0.4% | **CXCL13** | CXCL | chemokine (C-X-C motif) ligand 13 (B-cell chemoattractant) |
| **4** | 0.4% | **CCL27** | CCL | chemokine (C-C motif) ligand 27 |
| **4** | 0.4% | **CCL13** | CCL | chemokine (C-C motif) ligand 13 |
| **4** | 0.4% | **AGR2** | AGR | anterior gradient homolog 2 (Xenopus laevis) |
| **4** | 0.4% | **ADIPOQ** | ADIPO | adiponectin, C1Q and collagen domain containing |
| **4** | 0.4% | **WIF1** | WISP | WNT inhibitory factor 1 |
| **4** | 0.4% | **CXCL11** | CXCL | chemokine (C-X-C motif) ligand 11 |
| **3** | 0.3% | **Wnt10B** | Wnt | wingless-type MMTV integration site family, member 10B |
| **3** | 0.3% | **VTN** | VTN | vitronectin |
| **3** | 0.3% | **SOSTDC1** | SOST | sclerostin domain containing 1 |
| **3** | 0.3% | **SFTPB** | SFTP | surfactant, pulmonary-associated protein B |
| **3** | 0.3% | **SDF4** | SDF | stromal cell derived factor 4 |
| **3** | 0.3% | **RSPO1** | RSPO | R-spondin homolog (Xenopus laevis) |
| **3** | 0.3% | **PON3** | PON | paraoxonase 3 |
| **3** | 0.3% | **PNLIPRP3** | PNLIP | pancreatic lipase-related protein 3 |
| **3** | 0.3% | **NTF3** | NTF | neurotrophin 3 |
| **3** | 0.3% | **NOV** | IGFBP | nephroblastoma overexpressed gene |
| **3** | 0.3% | **NGFB** | NGFB | nerve growth factor (beta polypeptide) |
| **3** | 0.3% | **LCN1** | LCN | lipocalin 1 (tear prealbumin) |
| **3** | 0.3% | **KLK4** | KLK | kallikrein-related peptidase 4 |
| **3** | 0.3% | **IL25** | Interleukin | interleukin 25 |
| **3** | 0.3% | **IHH** | IHH | Indian hedgehog homolog (Drosophila) |
| **3** | 0.3% | **IGFBP4** | IGFBP | insulin-like growth factor binding protein 4 |
| **3** | 0.3% | **FGFBP1** | FGFBP | fibroblast growth factor binding protein 1 |
| **3** | 0.3% | **FGF18** | FGF | fibroblast growth factor 18 |
| **3** | 0.3% | **FGF13** | FGF | fibroblast growth factor 13 |
| **3** | 0.3% | **FGF11** | FGF | fibroblast growth factor 11 |
| **3** | 0.3% | **FCN3** | FCN | ficolin (collagen/fibrinogen domain containing) 3 (Hakata antigen) |
| **3** | 0.3% | **FCN2** | FCN | ficolin (collagen/fibrinogen domain containing lectin) 2 (hucolin) |
| **3** | 0.3% | **FAM3A** | FAM | family with sequence similarity 3, member A |
| **3** | 0.3% | **EBI3** | Interleukin | Epstein-Barr virus induced gene 3 |
| **3** | 0.3% | **DKKL1** | DKK | dickkopf-like 1 (soggy) |
| **3** | 0.3% | **DEFB112** | DEF | defensin, beta 112 |
| **3** | 0.3% | **CXCL9** | CXCL | chemokine (C-X-C motif) ligand 9 |
| **3** | 0.3% | **CXCL1** | CXCL | chemokine (C-X-C motif) ligand |
| **3** | 0.3% | **CRELD2** | CRELD | cysteine-rich with EGF-like domains 2 |
| **3** | 0.3% | **CHRDL2** | CHRDL | chordin-like 2 |
| **3** | 0.3% | **CCL23** | CCL | chemokine (C-C motif) ligand 23 |
| **3** | 0.3% | **BCMP11** | AGR | anterior gradient homolog 3 (Xenopus laevis) |
| **3** | 0.3% | **AHSG** | AHSG | alpha-2-HS-glycoprotein |
| **3** | 0.3% | **A1BG** | A1BG | alpha-1-B glycoprotein |
| **2** | 0.2% | **ZG16** | ZG | zymogen granule protein 16 |
| **2** | 0.2% | **UNQ467** | UNQ | keratinocyte differentiation-associated protein |
| **2** | 0.2% | **TPP1** | TPP | tripeptidyl peptidase I |
| **2** | 0.2% | **TGFB1** | TGFB | transforming growth factor, beta 1 |
| **2** | 0.2% | **SRPX2** | SRPX | sushi-repeat-containing protein, X-linked 2 |
| **2** | 0.2% | **SPOCK1** | SPARC | sparc/osteonectin, cwcv and kazal-like domains proteoglycan |
| **2** | 0.2% | **SERPINF2** | SERPIN | serpin peptidase inhibitor, clade F, member 2 |
| **2** | 0.2% | **SERPINA3** | SERPIN | serpin peptidase inhibitor, clade A, member 3 |
| **2** | 0.2% | **SCGB1D4** | SCGB | secretoglobin, family 1D, member 4 |
| **2** | 0.2% | **SCGB1D1** | SCGB | secretoglobin, family 1D, member 1 |
| **2** | 0.2% | **SCG5** | SCG | secretogranin V (7B2 protein) |
| **2** | 0.2% | **SCG3** | SCG | secretogranin III |
| **2** | 0.2% | **RSPO4** | RSPO | R-spondin family, member 4 |
| **2** | 0.2% | **RETN** | RET | resistin |
| **2** | 0.2% | **REG1A** | REG | regenerating islet-derived 1 alpha |
| **2** | 0.2% | **RCN3** | RCN | reticulocalbin 3, EF-hand calcium binding domain |
| **2** | 0.2% | **RBP4** | RBP | retinol binding protein 4, plasma |
| **2** | 0.2% | **PTN** | Interleukin | pleiotrophin (heparin binding growth factor 8) |
| **2** | 0.2% | **PRAP1** | PRAP | proline-rich acidic protein 1 |
| **2** | 0.2% | **PPGB** | Cath | cathepsin A |
| **2** | 0.2% | **PNLIPRP1** | PNLIP | pancreatic lipase-related protein 1 |
| **2** | 0.2% | **PLTP** | PLTP | phospholipid transfer protein |
| **2** | 0.2% | **OGN** | OGN | osteoglycin |
| **2** | 0.2% | **LUM** | LUM | lumican |
| **2** | 0.2% | **LOC348174** | LOC | secretory protein LOC348174 |
| **2** | 0.2% | **LECT2** | LECT | leukocyte cell-derived chemotaxin 2 |
| **2** | 0.2% | **KLK5** | KLK | kallikrein-related peptidase 5 |
| **2** | 0.2% | **KLK11** | KLK | kallikrein-related peptidase 11 |
| **2** | 0.2% | **KERA** | KERA | keratocan |
| **2** | 0.2% | **INHBA** | TGFB | inhibin, beta A |
| **2** | 0.2% | **IL3** | Interleukin | interleukin 3 (colony-stimulating factor, multiple) |
| **2** | 0.2% | **IL2** | Interleukin | interleukin 2 |
| **2** | 0.2% | **IL17B** | Interleukin | interleukin 17B |
| **2** | 0.2% | **IFNE1** | IFN | interferon epsilon 1 |
| **2** | 0.2% | **GIF** | GIF | gastric intrinsic factor (vitamin B synthesis) |
| **2** | 0.2% | **GDF5** | TGFB | growth differentiation factor 5 |
| **2** | 0.2% | **GDF3** | TGFB | growth differentiation factor 3 |
| **2** | 0.2% | **GDDR** | GKN | gastrokine 2 |
| **2** | 0.2% | **FGF6** | FGF | fibroblast growth factor 6 |
| **2** | 0.2% | **FGF22** | FGF | fibroblast growth factor 22 |
| **2** | 0.2% | **FGF20** | FGF | fibroblast growth factor 20 |
| **2** | 0.2% | **FGF19** | FGF | fibroblast growth factor 19 |
| **2** | 0.2% | **FGF12** | FGF | fibroblast growth factor 12 |
| **2** | 0.2% | **FAM3D** | FAM | family with sequence similarity 3, member D |
| **2** | 0.2% | **ESM1** | ESM | endothelial cell-specific molecule 1 |
| **2** | 0.2% | **EDIL3** | EDIL | EGF-like repeats and discoidin I-like domains 3 |
| **2** | 0.2% | **EBAG9** | EBAG | estrogen receptor binding site associated, antigen, 9 |
| **2** | 0.2% | **DKK4** | DKK | dickkopf homolog 4 (Xenopus laevis) |
| **2** | 0.2% | **DEFB119** | DEF | defensin, beta 119 |
| **2** | 0.2% | **DEFB104A** | DEF | defensin, beta 104A |
| **2** | 0.2% | **DEFA6** | DEF | defensin, alpha 6, Paneth cell-specific |
| **2** | 0.2% | **CXCL2** | CXCL | chemokine (C-X-C motif) ligand 2 |
| **2** | 0.2% | **CTGF** | IGFBP | connective tissue growth factor |
| **2** | 0.2% | **CLEC5A** | CLEC | C-type lectin domain family 5, member A |
| **2** | 0.2% | **CCL7** | CCL | chemokine (C-C motif) ligand 7 |
| **2** | 0.2% | **CCL15** | CCL | chemokine (C-C motif) ligand 15 |
| **2** | 0.2% | **CAMP** | CAMP | cathelicidin antimicrobial peptide |
| **2** | 0.2% | **CALU** | CALU | calumenin |
| **2** | 0.2% | **C1QTNF1** | C1Q | C1q and tumor necrosis factor related protein 1 |
| **2** | 0.2% | **BMP7** | TGFB | bone morphogenetic protein 7 |
| **2** | 0.2% | **BDNF** | BDNF | brain-derived neurotrophic factor |
| **2** | 0.2% | **AZGP1** | AZGP | alpha-2-glycoprotein 1, zinc-binding |
| **2** | 0.2% | **ANGPTL7** | ANG | angiopoietin-like 7 |
| **2** | 0.2% | **TCN1** | TCN | transcobalamin I (vitamin B12 binding protein, R binder family) |
| **2** | 0.2% | **F10** | F10 | coagulation factor X |
| **1** | 0.1% | **Wnt10A** | Wnt | wingless-type MMTV integration site family, member 10A |
| **1** | 0.1% | **Wnt8B** | Wnt | wingless-type MMTV integration site family, member 8B |
| **1** | 0.1% | **Wnt2B** | Wnt | wingless-type MMTV integration site family, member 2B |
| **1** | 0.1% | **Wnt1** | Wnt | wingless-type MMTV integration site family, member 1 |
| **1** | 0.1% | **WISP2** | WISP | WNT1 inducible signaling pathway protein 2 |
| **1** | 0.1% | **WFDC8** | WFDC | WAP four-disulfide core domain 8 |
| **1** | 0.1% | **WFDC5** | WFDC | WAP four-disulfide core domain 5 |
| **1** | 0.1% | **TNFAIP6** | TNF | tumor necrosis factor, alpha-induced protein 6 |
| **1** | 0.1% | **TLP19** | TLP | thioredoxin domain containing 12 (endoplasmic reticulum) |
| **1** | 0.1% | **TGFB2** | TGFB | transforming growth factor, beta 2 |
| **1** | 0.1% | **SPARCL1** | SPARC | SPARC-like 1 (mast9, hevin) |
| **1** | 0.1% | **SPARC** | SPARC | secreted protein, acidic, cysteine-rich (osteonectin) |
| **1** | 0.1% | **SPAG11** | SPAG | sperm associated antigen 11B |
| **1** | 0.1% | **SOST** | SOST | sclerosteosis |
| **1** | 0.1% | **Sfrp4** | SFRP | secreted frizzled-related protein 4 |
| **1** | 0.1% | **Sfrp2** | SFRP | secreted frizzled-related protein 2 |
| **1** | 0.1% | **SERPINA1** | SERPIN | serpin peptidase inhibitor, clade A, member 1 |
| **1** | 0.1% | **SCGB3A1** | SCGB | secretoglobin, family 3A, member 1 |
| **1** | 0.1% | **SAA4** | SAA | serum amyloid A4, constitutive |
| **1** | 0.1% | **RETNLB** | RET | resistin like beta |
| **1** | 0.1% | **REG3G** | REG | regenerating islet-derived 3 gamma |
| **1** | 0.1% | **PTX3** | PTX | pentraxin-related gene, rapidly induced by IL-1 beta |
| **1** | 0.1% | **PROK2** | PROK | prokineticin 2 |
| **1** | 0.1% | **PON2** | PON | paraoxonase 2 |
| **1** | 0.1% | **P4HA3** | P4HA | procollagen-proline, 2-oxoglutarate 4-dioxygenase  (proline 4-hydroxylase), alpha polypeptide III |
| **1** | 0.1% | **P4HA2** | P4HA | procollagen-proline, 2-oxoglutarate 4-dioxygenase  (proline 4-hydroxylase), alpha polypeptide II |
| **1** | 0.1% | **OTOR** | OTOR | otoraplin |
| **1** | 0.1% | **OIT3** | OIT | oncoprotein induced transcript 3 |
| **1** | 0.1% | **NTF5** | NTF | neurotrophin 4 |
| **1** | 0.1% | **nptx2** | PTX | neuronal pentraxin II |
| **1** | 0.1% | **NPTX1** | PTX | neuronal pentraxin I |
| **1** | 0.1% | **NPC2** | NPC | Niemann-Pick disease, type C2 |
| **1** | 0.1% | **MIA** | MIA | melanoma inhibitory activity |
| **1** | 0.1% | **LYPD1** | Ly-6 | LY6/PLAUR domain containing 1 |
| **1** | 0.1% | **LIPH** | LIP | lipase, member H |
| **1** | 0.1% | **Lefty2** | TGFB | left-right determination factor 2 |
| **1** | 0.1% | **LEAP2** | LEAP | liver expressed antimicrobial peptide 2 |
| **1** | 0.1% | **KLK7** | KLK | kallikrein-related peptidase 7 |
| **1** | 0.1% | **KLK13** | KLK | kallikrein-related peptidase 13 |
| **1** | 0.1% | **KLK10** | KLK | kallikrein-related peptidase 10 |
| **1** | 0.1% | **IL7** | Interleukin | interleukin 7 |
| **1** | 0.1% | **IL29** | Interleukin | interleukin 29 (interferon, lambda 1) |
| **1** | 0.1% | **IL26** | Interleukin | interleukin 26 |
| **1** | 0.1% | **IL24** | Interleukin | interleukin 24 |
| **1** | 0.1% | **IL17F** | Interleukin | interleukin 17F |
| **1** | 0.1% | **IL15** | Interleukin | interleukin 15 |
| **1** | 0.1% | **IL12A** | Interleukin | interleukin 12A |
| **1** | 0.1% | **IGFBP6** | IGFBP | insulin-like growth factor binding protein 6 |
| **1** | 0.1% | **IFNB1** | IFN | interferon, beta 1, fibroblast |
| **1** | 0.1% | **IFI30** | IFN | interferon, gamma-inducible protein 30 |
| **1** | 0.1% | **HPR** | HPR | haptoglobin-related protein |
| **1** | 0.1% | **HABP2** | HABP | hyaluronan binding protein 2 |
| **1** | 0.1% | **GREM2** | GREM | gremlin 2, cysteine knot superfamily, homolog (Xenopus laevis) |
| **1** | 0.1% | **GDF9** | TGFB | growth differentiation factor 9 |
| **1** | 0.1% | **GDF15** | TGFB | growth differentiation factor 15 |
| **1** | 0.1% | **FGF5** | FGF | fibroblast growth factor 5 |
| **1** | 0.1% | **FGF16** | FGF | fibroblast growth factor 16 |
| **1** | 0.1% | **DKK3** | DKK | dickkopf homolog 3 (Xenopus laevis) |
| **1** | 0.1% | **DEFB127** | DEF | defensin, beta 127 |
| **1** | 0.1% | **DEFB125** | DEF | defensin, beta 125 |
| **1** | 0.1% | **DEFB121** | DEF | defensin, beta 121 |
| **1** | 0.1% | **DEFB114** | DEF | defensin, beta 114 |
| **1** | 0.1% | **DEFB111** | DEF | defensin, beta 111 |
| **1** | 0.1% | **DEFB110** | DEF | defensin, beta 110 |
| **1** | 0.1% | **DEFB106A** | DEF | defensin, beta 106A |
| **1** | 0.1% | **CXCL10** | CXCL | chemokine (C-X-C motif) ligand 10 |
| **1** | 0.1% | **CST8** | CST | cystatin 8 (cystatin-related epididymal specific) |
| **1** | 0.1% | **CST7** | CST | cystatin F (leukocystatin) |
| **1** | 0.1% | **CST1** | CST | cystatin SN |
| **1** | 0.1% | **CRISP3** | CRISP | cysteine-rich secretory protein 3 |
| **1** | 0.1% | **CRISP2** | CRISP | cysteine-rich secretory protein 2 |
| **1** | 0.1% | **CRELD1** | CRELD | cysteine-rich with EGF-like domains 1 |
| **1** | 0.1% | **COLEC10** | CLEC | collectin sub-family member 10 (C-type lectin) |
| **1** | 0.1% | **CLU** | CLU | clusterin |
| **1** | 0.1% | **CLEC3A** | CLEC | C-type lectin domain family 3, member A |
| **1** | 0.1% | **CETP** | CETP | cholesteryl ester transfer protein, plasma |
| **1** | 0.1% | **CCL8** | CCL | chemokine (C-C motif) ligand 8 |
| **1** | 0.1% | **CCL26** | CCL | chemokine (C-C motif) ligand 26 |
| **1** | 0.1% | **CCL19** | CCL | chemokine (C-C motif) ligand 19 |
| **1** | 0.1% | **CCL14** | CCL | chemokine (C-C motif) ligand 14 |
| **1** | 0.1% | **C3orf9 / KTELC1** | KTEL | KTEL (Lys-Tyr-Glu-Leu) containing 1 |
| **1** | 0.1% | **C1QTNF3** | C1Q | C1q and tumor necrosis factor related protein 3 |
| **1** | 0.1% | **C1QTNF2** | C1Q | C1q and tumor necrosis factor related protein 2 |
| **1** | 0.1% | **BPIL1** | BPI | bactericidal/permeability-increasing protein-like 1 |
| **1** | 0.1% | **BPI** | BPI | bactericidal/permeability-increasing protein |
| **1** | 0.1% | **BMP5** | TGFB | bone morphogenetic protein 5 |
| **1** | 0.1% | **BMP3** | TGFB | bone morphogenetic protein 3 |
| **1** | 0.1% | **BGN** | BGN | biglycan |
| **1** | 0.1% | **APCS** | APCS | amyloid P component, serum |
| **1** | 0.1% | **ANGPT4** | ANGPT | angiopoietin 4 |
