# Supplementary material for: Systematic analysis of secreted proteins reveals synergism between IL6 and other proteins in soft agar growth of MCF10A cells
Source: Cell Biosci. 2011 Mar 25;1:13. doi: 10.1186/2045-3701-1-13 (PMC3125203; doi:10.1186/2045-3701-1-13)
Supplement: Additional file 4 — List of protein families recovered from screen colonies. The DNA recovered from the screen colonies was grouped in protein families, and the most prevalent protein families are listed here. As multiple protein family members may co-occur in a single colony, the number of colonies may be less than the number of inserts where the protein family is represented. Proteins that have no family members in this screen are listed as individual proteins. [file 2045-3701-1-13-S4.DOC]

| Additional table 3: List of protein families recovered from screen colonies | | | |
| --- | --- | --- | --- |
| **297 colonies total** | | |  |
| **total inserts** | **Total colonies** | **% of all colonies** | **Protein family** |
| **97** | **83** | **27.9%** | Interleukin |
| **97** | **88** | **29.5%** | WFDC |
| **78** | **72** | **24.2%** | FGF |
| **54** | **54** | **18.1%** | Wnt |
| **40** | **39** | **13.1%** | PON |
| **35** | **34** | **11.4%** | KLK |
| **33** | **28** | **9.4%** | TGFB |
| **21** | **19** | **6.4%** | CXCL |
| **21** | **21** | **7.0%** | DEF |
| **21** | **21** | **7.0%** | FGFBP |
| **26** | **26** | **8.7%** | TIMP |
| **19** | **19** | **6.4%** | CCL |
| **19** | **19** | **6.4%** | IGFBP |
| **18** | **18** | **6.0%** | RSPO |
| **16** | **15** | **5.0%** | DKK |
| **15** | **15** | **5.0%** | SAA |
| **14** | **14** | **4.7%** | PTHLH |
| **14** | **14** | **4.7%** | SFTP |
| **14** | **14** | **4.7%** | WISP |
| **13** | **13** | **4.4%** | APO |
| **13** | **13** | **4.4%** | Ly-6 |
| **13** | **13** | **4.4%** | PGLYRP |
| **12** | **12** | **4.0%** | MASP |
| **10** | **10** | **3.4%** | FAM |
| **10** | **10** | **3.4%** | IFN |
| **10** | **10** | **3.4%** | SPARC |
| **10** | **10** | **3.4%** | SUMF |
| **9** | **9** | **3.0%** | AZU |
| **9** | **9** | **3.0%** | C1Q |
| **7** | **7** | **2.3%** | Ly86 |
| **8** | **8** | **2.7%** | STC |
| **7** | **7** | **2.3%** | AGR |
| **7** | **6** | **2.0%** | lipase |
| **7** | **7** | **2.3%** | UTS |
| **6** | **6** | **2.0%** | FCN |
| **6** | **6** | **2.0%** | ORM |
| **6** | **6** | **2.0%** | SRPX |
| **6** | **6** | **2.0%** | RCN |
| **5** | **5** | **1.7%** | PNLIP |
| **5** | **5** | **1.7%** | SCGB |
| **5** | **5** | **1.7%** | SERPIN |
| **4** | **4** | **1.3%** | ADIPO |
| **4** | **4** | **1.3%** | CLEC |
| **4** | **4** | **1.3%** | CRELD |
| **4** | **4** | **1.3%** | FMOD |
| **4** | **4** | **1.3%** | NTF |
| **4** | **4** | **1.3%** | PI |
| **4** | **4** | **1.3%** | PLA1A |
| **4** | **4** | **1.3%** | SCG |
| **4** | **4** | **1.3%** | SOST |
| **4** | **4** | **1.3%** | TEX |
| **3** | **3** | **1.0%** | A1BG |
| **3** | **3** | **1.0%** | AHSG |
| **3** | **3** | **1.0%** | ANG |
| **3** | **3** | **1.0%** | CHRDL |
| **3** | **3** | **1.0%** | CST |
| **3** | **3** | **1.0%** | IHH |
| **3** | **3** | **1.0%** | LCN |
| **3** | **3** | **1.0%** | NGFB |
| **3** | **3** | **1.0%** | PTX |
| **3** | **3** | **1.0%** | REG |
| **3** | **3** | **1.0%** | RET |
| **3** | **3** | **1.0%** | SDF |
| **3** | **3** | **1.0%** | VTN |
| **2** | **2** | **0.7%** | AZGP |
| **2** | **2** | **0.7%** | BDNF |
| **2** | **2** | **0.7%** | BPI |
| **2** | **2** | **0.7%** | CALU |
| **2** | **2** | **0.7%** | CAMP |
| **2** | **2** | **0.7%** | Cath |
| **2** | **2** | **0.7%** | CRISP |
| **2** | **2** | **0.7%** | EBAG |
| **2** | **2** | **0.7%** | EDIL |
| **2** | **2** | **0.7%** | ESM |
| **2** | **2** | **0.7%** | F10 |
| **2** | **2** | **0.7%** | GIF |
| **2** | **2** | **0.7%** | GKN |
| **2** | **2** | **0.7%** | KERA |
| **2** | **2** | **0.7%** | LECT |
| **2** | **2** | **0.7%** | LOC |
| **2** | **2** | **0.7%** | LUM |
| **2** | **2** | **0.7%** | OGN |
| **2** | **2** | **0.7%** | P4HA |
| **2** | **2** | **0.7%** | PLTP |
| **2** | **2** | **0.7%** | PRAP |
| **2** | **2** | **0.7%** | RBP |
| **2** | **2** | **0.7%** | SFRP |
| **2** | **2** | **0.7%** | TCN |
| **2** | **2** | **0.7%** | TPP |
| **2** | **2** | **0.7%** | UNQ |
| **2** | **2** | **0.7%** | ZG |
| **1** | **1** | **0.3%** | APCS |
| **1** | **1** | **0.3%** | CETP |
| **1** | **1** | **0.3%** | CLU |
| **1** | **1** | **0.3%** | BGN |
| **1** | **1** | **0.3%** | GREM |
| **1** | **1** | **0.3%** | HABP |
| **1** | **1** | **0.3%** | HPR |
| **1** | **1** | **0.3%** | KTEL |
| **1** | **1** | **0.3%** | LEAP |
| **1** | **1** | **0.3%** | MIA |
| **1** | **1** | **0.3%** | NPC |
| **1** | **1** | **0.3%** | OIT |
| **1** | **1** | **0.3%** | OTOR |
| **1** | **1** | **0.3%** | PROK |
| **1** | **1** | **0.3%** | SPAG |
| **1** | **1** | **0.3%** | TLP |
| **1** | **1** | **0.3%** | TNFAIP |
