# Supplementary material for: Systematic analysis of secreted proteins reveals synergism between IL6 and other proteins in soft agar growth of MCF10A cells
Source: Cell Biosci. 2011 Mar 25;1:13. doi: 10.1186/2045-3701-1-13 (PMC3125203; doi:10.1186/2045-3701-1-13)
Supplement: Additional file 5 — DNA inserts in each colony. The actual DNA inserts recovered from each individual colony. [file 2045-3701-1-13-S5.DOC]

| **Additional table 4: DNA inserts in each individual colony** | |  |  |  |  |  |  |  |  |  |  |  |  |  |  |  |  |  |  |  |
| --- | --- | --- | --- | --- | --- | --- | --- | --- | --- | --- | --- | --- | --- | --- | --- | --- | --- | --- | --- | --- |
|  | |  |  |  |  |  |  |  |  |  |  |  |  |  |  |  |  |  |  |  |
| **Clones with 1 insert** | |  |  |  |  |  |  |  |  |  |  |  |  |  |  |  |  |  |  |  |
| **Clone label** | **Insert** |  |  |  |  |  |  |  |  |  |  |  |  |  |  |  |  |  |  |  |
| **P3/C1** | FGFBP2/KSP37 | |  |  |  |  |  |  |  |  |  |  |  |  |  |  |  |  |  |  |
| **P16/A3** | DKKL1 |  |  |  |  |  |  |  |  |  |  |  |  |  |  |  |  |  |  |  |
| **P11/B5** | FGF1 |  |  |  |  |  |  |  |  |  |  |  |  |  |  |  |  |  |  |  |
| **P5/B5** | FGF6 |  |  |  |  |  |  |  |  |  |  |  |  |  |  |  |  |  |  |  |
| **P16/A6** | FGF10 |  |  |  |  |  |  |  |  |  |  |  |  |  |  |  |  |  |  |  |
| **P6/C1** | FGF22 |  |  |  |  |  |  |  |  |  |  |  |  |  |  |  |  |  |  |  |
| **p3/a3** | Wnt3 |  |  |  |  |  |  |  |  |  |  |  |  |  |  |  |  |  |  |  |
| **p10/b2** | Wnt8A |  |  |  |  |  |  |  |  |  |  |  |  |  |  |  |  |  |  |  |
| **P7/C2** | MASP1 |  |  |  |  |  |  |  |  |  |  |  |  |  |  |  |  |  |  |  |
| **p13/c5** | IL9 |  |  |  |  |  |  |  |  |  |  |  |  |  |  |  |  |  |  |  |
| **P17/D5** | IL21 |  |  |  |  |  |  |  |  |  |  |  |  |  |  |  |  |  |  |  |
| **P16/B5** | IL2 |  |  |  |  |  |  |  |  |  |  |  |  |  |  |  |  |  |  |  |
| **p10/b3** | IL22 |  |  |  |  |  |  |  |  |  |  |  |  |  |  |  |  |  |  |  |
| **p4/d3** | IL22 |  |  |  |  |  |  |  |  |  |  |  |  |  |  |  |  |  |  |  |
| **P4/A6** | IL31 |  |  |  |  |  |  |  |  |  |  |  |  |  |  |  |  |  |  |  |
| **p7/a4** | WFDC7 |  |  |  |  |  |  |  |  |  |  |  |  |  |  |  |  |  |  |  |
| **P7/A2** | WFDC13 |  |  |  |  |  |  |  |  |  |  |  |  |  |  |  |  |  |  |  |
| **P13/A4** | WFDC14 |  |  |  |  |  |  |  |  |  |  |  |  |  |  |  |  |  |  |  |
| **p9/d2/a** | CXCL7 |  |  |  |  |  |  |  |  |  |  |  |  |  |  |  |  |  |  |  |
| **P10/C5/B** | DEFB130 |  |  |  |  |  |  |  |  |  |  |  |  |  |  |  |  |  |  |  |
| **P17/B3B** | LY86 |  |  |  |  |  |  |  |  |  |  |  |  |  |  |  |  |  |  |  |
| **P15/A3A** | UTS2 |  |  |  |  |  |  |  |  |  |  |  |  |  |  |  |  |  |  |  |
| **P8/C4** | LYNX1 |  |  |  |  |  |  |  |  |  |  |  |  |  |  |  |  |  |  |  |
| **P16/D4** | CST1 |  |  |  |  |  |  |  |  |  |  |  |  |  |  |  |  |  |  |  |
| **P14/B5** | CST7 |  |  |  |  |  |  |  |  |  |  |  |  |  |  |  |  |  |  |  |
| **P1/C1** | KLK6 |  |  |  |  |  |  |  |  |  |  |  |  |  |  |  |  |  |  |  |
| **p7/a3** | PON1 |  |  |  |  |  |  |  |  |  |  |  |  |  |  |  |  |  |  |  |
| **P16/D5A** | PON1 |  |  |  |  |  |  |  |  |  |  |  |  |  |  |  |  |  |  |  |
| **P16/D5A/2** | PON1 |  |  |  |  |  |  |  |  |  |  |  |  |  |  |  |  |  |  |  |
| **P5/B3** | SCGB1D4 |  |  |  |  |  |  |  |  |  |  |  |  |  |  |  |  |  |  |  |
| **P16/A5** | calu |  |  |  |  |  |  |  |  |  |  |  |  |  |  |  |  |  |  |  |
| **P12/A3** | SUMF1 |  |  |  |  |  |  |  |  |  |  |  |  |  |  |  |  |  |  |  |

| **Clones with 2 inserts** | |  |  |  |  |  |  |  |  |  |  |  |  |  |  |  |  |  |  |  |
| --- | --- | --- | --- | --- | --- | --- | --- | --- | --- | --- | --- | --- | --- | --- | --- | --- | --- | --- | --- | --- |
| **Clone label** | **Insert1** | **Insert2** |  |  |  |  |  |  |  |  |  |  |  |  |  |  |  |  |  |  |
| **P6/A5** | IGFBP4 | WIF1 |  |  |  |  |  |  |  |  |  |  |  |  |  |  |  |  |  |  |
| **P10/B4** | IGFBP5 | PON1 |  |  |  |  |  |  |  |  |  |  |  |  |  |  |  |  |  |  |
| **P10/C5** | SOSTDC1 | PON1 |  |  |  |  |  |  |  |  |  |  |  |  |  |  |  |  |  |  |
| **P12/C6** | sostdc1 | cxcl11 |  |  |  |  |  |  |  |  |  |  |  |  |  |  |  |  |  |  |
| **P6/A6** | FGFBP1 | WFDC13 |  |  |  |  |  |  |  |  |  |  |  |  |  |  |  |  |  |  |
| **P4/D1** | FGFBP2/KSP37 | Wnt3 |  |  |  |  |  |  |  |  |  |  |  |  |  |  |  |  |  |  |
| **P3/C1/B** | FGFBP2/KSP37 | EBAG9 |  |  |  |  |  |  |  |  |  |  |  |  |  |  |  |  |  |  |
| **P10/C1** | DKK1 | LIPH |  |  |  |  |  |  |  |  |  |  |  |  |  |  |  |  |  |  |
| **P1/C3** | DKK4 | FCN2 |  |  |  |  |  |  |  |  |  |  |  |  |  |  |  |  |  |  |
| **P14/B3** | CRELD2 | PON1 |  |  |  |  |  |  |  |  |  |  |  |  |  |  |  |  |  |  |
| **P15/A2** | RSPO1 | LYNX1 |  |  |  |  |  |  |  |  |  |  |  |  |  |  |  |  |  |  |
| **P8/D1** | FAM3A | AZU1 |  |  |  |  |  |  |  |  |  |  |  |  |  |  |  |  |  |  |
| **P13/C6** | FAM3A | KLK6 |  |  |  |  |  |  |  |  |  |  |  |  |  |  |  |  |  |  |
| **P7/C6** | FAM3D | NODAL |  |  |  |  |  |  |  |  |  |  |  |  |  |  |  |  |  |  |
| **P7/C5** | PTX3 | SERPINA3 |  |  |  |  |  |  |  |  |  |  |  |  |  |  |  |  |  |  |
| **P12/B6** | GDF8 | TPP1 |  |  |  |  |  |  |  |  |  |  |  |  |  |  |  |  |  |  |
| **P15/B4** | GDF8 | TGFB1 |  |  |  |  |  |  |  |  |  |  |  |  |  |  |  |  |  |  |
| **p6/a1** | FGF5 | IL6 |  |  |  |  |  |  |  |  |  |  |  |  |  |  |  |  |  |  |
| **P9/C6** | FGF9 | CRISP2 |  |  |  |  |  |  |  |  |  |  |  |  |  |  |  |  |  |  |
| **P9/A1** | FGF9 | WFDC13 |  |  |  |  |  |  |  |  |  |  |  |  |  |  |  |  |  |  |
| **P13/C4** | FGF10 | WFDC13 |  |  |  |  |  |  |  |  |  |  |  |  |  |  |  |  |  |  |
| **P6/C4** | FGF10 | PGLYRP1 |  |  |  |  |  |  |  |  |  |  |  |  |  |  |  |  |  |  |
| **P11/D1B** | FGF11 | WFDC14 |  |  |  |  |  |  |  |  |  |  |  |  |  |  |  |  |  |  |
| **P6/B2** | FGF14 | KLK14 |  |  |  |  |  |  |  |  |  |  |  |  |  |  |  |  |  |  |
| **P2/C4** | Wnt3 | IL21 |  |  |  |  |  |  |  |  |  |  |  |  |  |  |  |  |  |  |
| **P15/b2** | Wnt3 | WFDC11 |  |  |  |  |  |  |  |  |  |  |  |  |  |  |  |  |  |  |
| **P10/A3A** | Wnt3 | LYNX1 |  |  |  |  |  |  |  |  |  |  |  |  |  |  |  |  |  |  |
| **P3/B1/B** | Wnt3 | AZGP1 |  |  |  |  |  |  |  |  |  |  |  |  |  |  |  |  |  |  |
| **P6/B1** | Wnt9A | MASP2 |  |  |  |  |  |  |  |  |  |  |  |  |  |  |  |  |  |  |
| **P6/C1/B** | C1QTNF2 | CCL13 |  |  |  |  |  |  |  |  |  |  |  |  |  |  |  |  |  |  |
| **P12/A4** | FCN2 | SAA1 |  |  |  |  |  |  |  |  |  |  |  |  |  |  |  |  |  |  |
| **P8/B5** | MASP2 | PON1 |  |  |  |  |  |  |  |  |  |  |  |  |  |  |  |  |  |  |
| **P6/A3A** | IL21 | IL20 |  |  |  |  |  |  |  |  |  |  |  |  |  |  |  |  |  |  |
| **P1/A6** | IL17 | PON1 |  |  |  |  |  |  |  |  |  |  |  |  |  |  |  |  |  |  |
| **P5/B1** | IL12B | IL20 |  |  |  |  |  |  |  |  |  |  |  |  |  |  |  |  |  |  |
| **p5/c2c** | PTN | APOD |  |  |  |  |  |  |  |  |  |  |  |  |  |  |  |  |  |  |
| **P12/B1** | IL6 | PON1 |  |  |  |  |  |  |  |  |  |  |  |  |  |  |  |  |  |  |
| **P9/B6** | IL6 | PI15 |  |  |  |  |  |  |  |  |  |  |  |  |  |  |  |  |  |  |
| **P12/A1A** | IL6 | LY86 |  |  |  |  |  |  |  |  |  |  |  |  |  |  |  |  |  |  |
| **P7/B4** | IL6 | WFDC13 |  |  |  |  |  |  |  |  |  |  |  |  |  |  |  |  |  |  |
| **P1/A4** | IL22 | WFDC13 |  |  |  |  |  |  |  |  |  |  |  |  |  |  |  |  |  |  |
| **P2/A3** | IL22 | AHSG |  |  |  |  |  |  |  |  |  |  |  |  |  |  |  |  |  |  |
| **p9/b4** | IL27 | WFDC13 |  |  |  |  |  |  |  |  |  |  |  |  |  |  |  |  |  |  |
| **P4/C2** | IL31 | APOH |  |  |  |  |  |  |  |  |  |  |  |  |  |  |  |  |  |  |
| **P11/B1** | AGR2 | ORM1 |  |  |  |  |  |  |  |  |  |  |  |  |  |  |  |  |  |  |
| **p15/c5** | TLP19 | WFDC13 |  |  |  |  |  |  |  |  |  |  |  |  |  |  |  |  |  |  |
| **P16/D2** | WFDC13 | CXCL7 |  |  |  |  |  |  |  |  |  |  |  |  |  |  |  |  |  |  |
| **P17/B3A** | WFDC13 | NTF5 |  |  |  |  |  |  |  |  |  |  |  |  |  |  |  |  |  |  |
| **P5/C4** | WFDC13 | KLK14 |  |  |  |  |  |  |  |  |  |  |  |  |  |  |  |  |  |  |
| **P12/C3** | IFNA1 | F10 |  |  |  |  |  |  |  |  |  |  |  |  |  |  |  |  |  |  |
| **p6/c5-b** | CHRDL2 | APOD |  |  |  |  |  |  |  |  |  |  |  |  |  |  |  |  |  |  |
| **P9/B5** | CXCL2 | KLK6 |  |  |  |  |  |  |  |  |  |  |  |  |  |  |  |  |  |  |
| **P11/A6** | CXCL9 | PON3 |  |  |  |  |  |  |  |  |  |  |  |  |  |  |  |  |  |  |
| **P16/C2** | CXCL9 | TEX264 |  |  |  |  |  |  |  |  |  |  |  |  |  |  |  |  |  |  |
| **P12/D5** | CXCL11 | HABP2 |  |  |  |  |  |  |  |  |  |  |  |  |  |  |  |  |  |  |
| **P11/B3** | CXCL13 | AZU1 |  |  |  |  |  |  |  |  |  |  |  |  |  |  |  |  |  |  |
| **P2/B4** | CCL23 | BGN |  |  |  |  |  |  |  |  |  |  |  |  |  |  |  |  |  |  |
| **P15/d3** | SPAG11 | IFI30 |  |  |  |  |  |  |  |  |  |  |  |  |  |  |  |  |  |  |
| **P14/C3** | DEFB119 | KLK3 |  |  |  |  |  |  |  |  |  |  |  |  |  |  |  |  |  |  |
| **P10/B1** | DEFB121 | KLK11 |  |  |  |  |  |  |  |  |  |  |  |  |  |  |  |  |  |  |
| **P15/B1** | UNQ467 | PON1 |  |  |  |  |  |  |  |  |  |  |  |  |  |  |  |  |  |  |
| **P15/B5** | RETN | KLK2 |  |  |  |  |  |  |  |  |  |  |  |  |  |  |  |  |  |  |
| **P17/B6C** | NGFB | LUM |  |  |  |  |  |  |  |  |  |  |  |  |  |  |  |  |  |  |
| **P7/B5** | TGFB1 | AZU1 |  |  |  |  |  |  |  |  |  |  |  |  |  |  |  |  |  |  |
| **P6/B3** | TIMP1 | WFDC13 |  |  |  |  |  |  |  |  |  |  |  |  |  |  |  |  |  |  |
| **P14/A4** | TIMP4 | ORM1 |  |  |  |  |  |  |  |  |  |  |  |  |  |  |  |  |  |  |
| **P9/D1** | CAMP | FMOD |  |  |  |  |  |  |  |  |  |  |  |  |  |  |  |  |  |  |
| **P13/B2** | AHSG | PON1 |  |  |  |  |  |  |  |  |  |  |  |  |  |  |  |  |  |  |
| **P14/C1B** | KLK3 | SCG5 |  |  |  |  |  |  |  |  |  |  |  |  |  |  |  |  |  |  |
| **P17/A5** | KLK3 | PTHLH |  |  |  |  |  |  |  |  |  |  |  |  |  |  |  |  |  |  |
| **P17/D3** | KLK6 | IGFALS |  |  |  |  |  |  |  |  |  |  |  |  |  |  |  |  |  |  |
| **P2/B1** | KLK7 | APOD |  |  |  |  |  |  |  |  |  |  |  |  |  |  |  |  |  |  |
| **P16/B2B** | PON1 | STC1 |  |  |  |  |  |  |  |  |  |  |  |  |  |  |  |  |  |  |
| **P9/C5** | PON1 | WFDC13 |  |  |  |  |  |  |  |  |  |  |  |  |  |  |  |  |  |  |
| **P9/B2** | PON1 | PNLIPRP3 |  |  |  |  |  |  |  |  |  |  |  |  |  |  |  |  |  |  |
| **P17/B5B** | PON3 | PTHLH |  |  |  |  |  |  |  |  |  |  |  |  |  |  |  |  |  |  |
| **P17/C4** | SPARC | PLA1A |  |  |  |  |  |  |  |  |  |  |  |  |  |  |  |  |  |  |
| **P9/C1** | ZG16 | PLA1A |  |  |  |  |  |  |  |  |  |  |  |  |  |  |  |  |  |  |

| **Clones with 3inserts** | |  |  |  |  |  |  |  |  |  |  |  |  |  |  |  |  |  |  |  |
| --- | --- | --- | --- | --- | --- | --- | --- | --- | --- | --- | --- | --- | --- | --- | --- | --- | --- | --- | --- | --- |
| **Clone label** | **Insert1** | **Insert2** | **Insert3** |  |  |  |  |  |  |  |  |  |  |  |  |  |  |  |  |  |
| **P7/B3** | IGFBP5 | IL31 | WFDC13 |  |  |  |  |  |  |  |  |  |  |  |  |  |  |  |  |  |
| **P2/C2** | IGFBP5 | GDF3 | WFDC4/SLPI | |  |  |  |  |  |  |  |  |  |  |  |  |  |  |  |  |
| **P12/A2** | NOV | GDF15 | CXCL1 |  |  |  |  |  |  |  |  |  |  |  |  |  |  |  |  |  |
| **P13/A1** | FGFBP2/KSP37 | ANGPTL7 | TIMP1 |  |  |  |  |  |  |  |  |  |  |  |  |  |  |  |  |  |
| **P11/C2** | FGFBP2/KSP37 | FGF10 | LYNX1 |  |  |  |  |  |  |  |  |  |  |  |  |  |  |  |  |  |
| **P13/B4** | FGFBP2/KSP37 | WFDC11 | SCGB1D4 |  |  |  |  |  |  |  |  |  |  |  |  |  |  |  |  |  |
| **P10/B5** | FGFBP2/KSP37 | FGF3 | ADIPOQ |  |  |  |  |  |  |  |  |  |  |  |  |  |  |  |  |  |
| **P15/C4** | FGFBP2/KSP37 | Wnt3 | NODAL |  |  |  |  |  |  |  |  |  |  |  |  |  |  |  |  |  |
| **p11/b6** | FGFBP2/KSP37 | Wnt1 | UNQ467 |  |  |  |  |  |  |  |  |  |  |  |  |  |  |  |  |  |
| **P16/A4** | DKK1 | TEX264 | PTHLH |  |  |  |  |  |  |  |  |  |  |  |  |  |  |  |  |  |
| **P12/D3** | DKK1 | P4HA3 | PNLIPRP1 |  |  |  |  |  |  |  |  |  |  |  |  |  |  |  |  |  |
| **P3/A1** | DKK2 | GDF3 | BPIL1 |  |  |  |  |  |  |  |  |  |  |  |  |  |  |  |  |  |
| **P13/A5** | DKK2 | OIT3 | SAA1 |  |  |  |  |  |  |  |  |  |  |  |  |  |  |  |  |  |
| **P15/C5/B** | RSPO3 | SFTPB | OTOR |  |  |  |  |  |  |  |  |  |  |  |  |  |  |  |  |  |
| **P5/D5** | RSPO3 | BMP7 | Wnt2 |  |  |  |  |  |  |  |  |  |  |  |  |  |  |  |  |  |
| **P14/C4** | RSPO4 | FGF9 | CCL8 |  |  |  |  |  |  |  |  |  |  |  |  |  |  |  |  |  |
| **P5/C6** | FAM3B | IL6 | CCL15 |  |  |  |  |  |  |  |  |  |  |  |  |  |  |  |  |  |
| **P1/A2** | NPTX1 | FCN3 | SFTPA2 |  |  |  |  |  |  |  |  |  |  |  |  |  |  |  |  |  |
| **P16/B3** | GDF8 | WFDC13 | SPON2 |  |  |  |  |  |  |  |  |  |  |  |  |  |  |  |  |  |
| **P2/B5** | FGF1 | WFDC13 | CCL26 |  |  |  |  |  |  |  |  |  |  |  |  |  |  |  |  |  |
| **P9/A3** | FGF1 | Wnt2 | SFTPB |  |  |  |  |  |  |  |  |  |  |  |  |  |  |  |  |  |
| **P4/B5** | FGF3 | lynx1 | WFDC13 |  |  |  |  |  |  |  |  |  |  |  |  |  |  |  |  |  |
| **P12/A5** | FGF3 | FCN2 | SAA1 |  |  |  |  |  |  |  |  |  |  |  |  |  |  |  |  |  |
| **P8/A4** | FGF4 | Wnt3 | IFNA1 |  |  |  |  |  |  |  |  |  |  |  |  |  |  |  |  |  |
| **P12/B5** | FGF4 | ADIPOQ | WFDC4 |  |  |  |  |  |  |  |  |  |  |  |  |  |  |  |  |  |
| **P8/C6** | FGF10 | IL17B | PON1 |  |  |  |  |  |  |  |  |  |  |  |  |  |  |  |  |  |
| **p15/b3** | FGF10 | WFDC13 | TIMP3 |  |  |  |  |  |  |  |  |  |  |  |  |  |  |  |  |  |
| **P13/B5** | FGF13 | SFTPB | PON1 |  |  |  |  |  |  |  |  |  |  |  |  |  |  |  |  |  |
| **P16/A1** | FGF14 | IL31 | PON1 |  |  |  |  |  |  |  |  |  |  |  |  |  |  |  |  |  |
| **P1/A3** | FGF14 | Wnt3 | TIMP3 |  |  |  |  |  |  |  |  |  |  |  |  |  |  |  |  |  |
| **P12/D4B** | FGF14 | Wnt2 | DEFB127 |  |  |  |  |  |  |  |  |  |  |  |  |  |  |  |  |  |
| **p5/a2** | FGF18 | BDNF | NPC2 |  |  |  |  |  |  |  |  |  |  |  |  |  |  |  |  |  |
| **P13/A3** | FGF18 | INHBA | UTS2 |  |  |  |  |  |  |  |  |  |  |  |  |  |  |  |  |  |
| **P17/D6** | FGF20 | IL9 | SPON2 |  |  |  |  |  |  |  |  |  |  |  |  |  |  |  |  |  |
| **p5/c3B** | FGF22 | CHRDL2 | SAA1 |  |  |  |  |  |  |  |  |  |  |  |  |  |  |  |  |  |
| **P17/D2** | Wnt2 | LYPD1 | SUMF1 |  |  |  |  |  |  |  |  |  |  |  |  |  |  |  |  |  |
| **P11/C6** | Wnt2B | IL21 | IL17B |  |  |  |  |  |  |  |  |  |  |  |  |  |  |  |  |  |
| **P5/C1** | Wnt3 | SFTPA2 | UTS2 |  |  |  |  |  |  |  |  |  |  |  |  |  |  |  |  |  |
| **P10/A1** | Wnt3 | WFDC13 | PGLYRP1 |  |  |  |  |  |  |  |  |  |  |  |  |  |  |  |  |  |
| **p5/b4** | Wnt3 | WFDC13 | CCL23 |  |  |  |  |  |  |  |  |  |  |  |  |  |  |  |  |  |
| **P3/B5** | Wnt3 | CCL14 | SDF4 |  |  |  |  |  |  |  |  |  |  |  |  |  |  |  |  |  |
| **P2/B2** | Wnt3 | WFDC8 | STC2 |  |  |  |  |  |  |  |  |  |  |  |  |  |  |  |  |  |
| **P5/A5** | Wnt3 | IHH | TIMP4 |  |  |  |  |  |  |  |  |  |  |  |  |  |  |  |  |  |
| **P4/B4** | Wnt9a | MASP2 | SAA1 |  |  |  |  |  |  |  |  |  |  |  |  |  |  |  |  |  |
| **P8/C5** | Wnt10B | BCMP11 | SRPX2 |  |  |  |  |  |  |  |  |  |  |  |  |  |  |  |  |  |
| **P3/C3** | ADIPOQ | STC2 | LPL |  |  |  |  |  |  |  |  |  |  |  |  |  |  |  |  |  |
| **P16/C6B** | C1QTNF6 | WFDC13 | PRAP1 |  |  |  |  |  |  |  |  |  |  |  |  |  |  |  |  |  |
| **P8/B3** | LOC348174 | MASP1 | WFDC13 |  |  |  |  |  |  |  |  |  |  |  |  |  |  |  |  |  |
| **P17/A6** | LOC348174 | WFDC14 | KLK4 |  |  |  |  |  |  |  |  |  |  |  |  |  |  |  |  |  |
| **P8/B2** | MASP2 | CCL15 | HPR |  |  |  |  |  |  |  |  |  |  |  |  |  |  |  |  |  |
| **P10/A3B** | IL21 | WFDC13 | PON1 |  |  |  |  |  |  |  |  |  |  |  |  |  |  |  |  |  |
| **P3/C4** | IL17 | KLK5 | SRPX |  |  |  |  |  |  |  |  |  |  |  |  |  |  |  |  |  |
| **P9/A4** | IL12B | KLK5 | SUMF1 |  |  |  |  |  |  |  |  |  |  |  |  |  |  |  |  |  |
| **P9/B1** | IL6 | WFDC14 | PON1 |  |  |  |  |  |  |  |  |  |  |  |  |  |  |  |  |  |
| **P8/A5** | IL22 | WFDC13 | IGFALS |  |  |  |  |  |  |  |  |  |  |  |  |  |  |  |  |  |
| **P17/C2** | IL27 | WFDC13 | SERPINA1 |  |  |  |  |  |  |  |  |  |  |  |  |  |  |  |  |  |
| **P5/C2** | IL27 | KLK13 | SCG5 |  |  |  |  |  |  |  |  |  |  |  |  |  |  |  |  |  |
| **P13/B6** | wfdc13 | ESM1 | PTHLH |  |  |  |  |  |  |  |  |  |  |  |  |  |  |  |  |  |
| **P5/C3** | WFDC13 | IFNB1 | SAA1 |  |  |  |  |  |  |  |  |  |  |  |  |  |  |  |  |  |
| **P7/C4** | WFDC13 | LCN1 | PTHLH |  |  |  |  |  |  |  |  |  |  |  |  |  |  |  |  |  |
| **P16/C3B** | WFDC13 | AZU1 | FMOD |  |  |  |  |  |  |  |  |  |  |  |  |  |  |  |  |  |
| **P16/B4** | WFDC13 | KERA | SAA1 |  |  |  |  |  |  |  |  |  |  |  |  |  |  |  |  |  |
| **P12/C5** | WFDC13 | ORM1 | PTHLH |  |  |  |  |  |  |  |  |  |  |  |  |  |  |  |  |  |
| **P2/C3** | WFDC14 | DEFA6 | SERPINF2 |  |  |  |  |  |  |  |  |  |  |  |  |  |  |  |  |  |
| **P6/C5** | CHRDL2 | APOD | EDIL3 |  |  |  |  |  |  |  |  |  |  |  |  |  |  |  |  |  |
| **P2/B6** | CXCL7 | DEFB111 | CALU |  |  |  |  |  |  |  |  |  |  |  |  |  |  |  |  |  |
| **P5/B6** | PLTP | SERPINF2 | OGN |  |  |  |  |  |  |  |  |  |  |  |  |  |  |  |  |  |
| **P16/B1** | KLK3 | stc2 | orm1 |  |  |  |  |  |  |  |  |  |  |  |  |  |  |  |  |  |
|  |  |  |  |  |  |  |  |  |  |  |  |  |  |  |  |  |  |  |  |  |

| **Clones with 4 inserts** | |  |  |  |  |  |  |  |  |  |  |  |  |  |  |  |  |  |  |  |
| --- | --- | --- | --- | --- | --- | --- | --- | --- | --- | --- | --- | --- | --- | --- | --- | --- | --- | --- | --- | --- |
| **Clone label** | **Insert1** | **Insert2** | **Insert3** | **Insert4** |  |  |  |  |  |  |  |  |  |  |  |  |  |  |  |  |
| **P6/B4** | IGFBP4 | FGF23 | Wnt8B | DEFB125 |  |  |  |  |  |  |  |  |  |  |  |  |  |  |  |  |
| **P1/B1A** | IGFBP6 | APCS | IL20 | PTHLH |  |  |  |  |  |  |  |  |  |  |  |  |  |  |  |  |
| **p6/c2** | WISP1 | FGF14 | IL22 | BCMP11 |  |  |  |  |  |  |  |  |  |  |  |  |  |  |  |  |
| **P4/B6** | WISP2 | FGF10 | Lefty2 | PLA1A |  |  |  |  |  |  |  |  |  |  |  |  |  |  |  |  |
| **P4/A3** | WISP3 | Wnt3 | KLK11 | WFDC13 |  |  |  |  |  |  |  |  |  |  |  |  |  |  |  |  |
| **P14/A5** | WISP3 | WFDC13 | TIMP1 | EDIL3 |  |  |  |  |  |  |  |  |  |  |  |  |  |  |  |  |
| **P17/D4** | WIF1 | SFTPA2 | IL29 | SUMF1 |  |  |  |  |  |  |  |  |  |  |  |  |  |  |  |  |
| **p4/c6** | WIF1 | EBI3 | Wfdc13 | IGFALS |  |  |  |  |  |  |  |  |  |  |  |  |  |  |  |  |
| **P3/A4** | SOST | FGF14 | PON1 | PON2 |  |  |  |  |  |  |  |  |  |  |  |  |  |  |  |  |
| **P4/A2** | FGFBP1 | FGF19 | PLA1A | LPL |  |  |  |  |  |  |  |  |  |  |  |  |  |  |  |  |
| **P2/A6** | FGFBP2/KSP37 | FGF14 | Wnt3 | SPON2 |  |  |  |  |  |  |  |  |  |  |  |  |  |  |  |  |
| **p11/a2B** | FGFBP2/KSP37 | Wfdc13 | CXCL1 | SCG3 |  |  |  |  |  |  |  |  |  |  |  |  |  |  |  |  |
| **P4/B1** | FGFBP2/KSP37 | C1QTNF6 | CCL27 | ESM1 |  |  |  |  |  |  |  |  |  |  |  |  |  |  |  |  |
| **P8/A2** | FGFBP2/KSP37 | IL6 | UTS2 | SMOC1 |  |  |  |  |  |  |  |  |  |  |  |  |  |  |  |  |
| **P11/C5** | Sfrp2 | FGF1 | Wnt3 | WFDC13 |  |  |  |  |  |  |  |  |  |  |  |  |  |  |  |  |
| **P13/A6** | DKK1 | Wnt9a | CCL13 | IGFALS |  |  |  |  |  |  |  |  |  |  |  |  |  |  |  |  |
| **P7/D3** | DKK1 | IL21 | TEX264 | PON3 |  |  |  |  |  |  |  |  |  |  |  |  |  |  |  |  |
| **P6/B6** | DKK3 | DKKL1 | MDK | IL22 |  |  |  |  |  |  |  |  |  |  |  |  |  |  |  |  |
| **P12/B3** | CRELD2 | BMP5 | WFDC7 | LPL |  |  |  |  |  |  |  |  |  |  |  |  |  |  |  |  |
| **P14/C2** | CRELD2 | WFDC13 | IFNE1 | SRPX |  |  |  |  |  |  |  |  |  |  |  |  |  |  |  |  |
| **P5/A1B** | RSPO3 | FAM3B | IL12B | CRISP3 |  |  |  |  |  |  |  |  |  |  |  |  |  |  |  |  |
| **P5/B2** | RSPO3 | SFTPA2 | ORM1 | DEFB112 |  |  |  |  |  |  |  |  |  |  |  |  |  |  |  |  |
| **P12/C1** | RSPO3 | WFDC11 | WFDC13 | TIMP2 |  |  |  |  |  |  |  |  |  |  |  |  |  |  |  |  |
| **P4/B2** | RSPO3 | PLTP | NTF3 | SAA1 |  |  |  |  |  |  |  |  |  |  |  |  |  |  |  |  |
| **P2/A2** | RSPO4 | GDF9 | SFTPC | C3orf9 / KTELC1 | |  |  |  |  |  |  |  |  |  |  |  |  |  |  |  |
| **P9/B2/B** | ANGPT4 | PTN | IL2 | BDNF |  |  |  |  |  |  |  |  |  |  |  |  |  |  |  |  |
| **P16/D3** | FAM3B | IL31 | DEFB119 | SPOCK1 |  |  |  |  |  |  |  |  |  |  |  |  |  |  |  |  |
| **P2/D2** | FAM3D | Wnt3 | STC1 | LPL |  |  |  |  |  |  |  |  |  |  |  |  |  |  |  |  |
| **P16/A2** | nptx2 | SFTPA2 | IL6 | STC1 |  |  |  |  |  |  |  |  |  |  |  |  |  |  |  |  |
| **P17/B6A** | GDF5 | WFDC4 | WFDC13 | RCN2 |  |  |  |  |  |  |  |  |  |  |  |  |  |  |  |  |
| **P3/B6** | GDF5 | C1QTNF6 | WFDC13 | NGFB |  |  |  |  |  |  |  |  |  |  |  |  |  |  |  |  |
| **P10/A5** | GDF8 | IL12B | DEFB130 | VTN |  |  |  |  |  |  |  |  |  |  |  |  |  |  |  |  |
| **P14/B1** | BMP7 | FGF10 | NTF3 | LPL |  |  |  |  |  |  |  |  |  |  |  |  |  |  |  |  |
| **P11/A5** | FGF1 | IL22 | CXCL10 | TIMP3 |  |  |  |  |  |  |  |  |  |  |  |  |  |  |  |  |
| **P16/C5** | FGF1 | FGF23 | Wnt3 | RCN3 |  |  |  |  |  |  |  |  |  |  |  |  |  |  |  |  |
| **P2/C6** | FGF4 | FGF10 | PGLYRP3 | SERPINA3 |  |  |  |  |  |  |  |  |  |  |  |  |  |  |  |  |
| **P10/C4** | FGF9 | LECT2 | TPP1 | LPL |  |  |  |  |  |  |  |  |  |  |  |  |  |  |  |  |
| **P17/B1** | FGF10 | IL3 | STC1 | GIF |  |  |  |  |  |  |  |  |  |  |  |  |  |  |  |  |
| **P1/B3** | FGF10 | GDDR | WFDC13 | CCL7 |  |  |  |  |  |  |  |  |  |  |  |  |  |  |  |  |
| **P14/B4** | FGF12 | FGF19 | Wnt3 | WFDC13 |  |  |  |  |  |  |  |  |  |  |  |  |  |  |  |  |
| **P7/C1** | FGF14 | MASP2 | IL21 | IL22 |  |  |  |  |  |  |  |  |  |  |  |  |  |  |  |  |
| **P8/C2** | FGF14 | TEX264 | SPOCK1 | FMOD |  |  |  |  |  |  |  |  |  |  |  |  |  |  |  |  |
| **P2/A4** | FGF20 | Wnt8A | COLEC10 | WFDC13 |  |  |  |  |  |  |  |  |  |  |  |  |  |  |  |  |
| **P8/C3** | Wnt2 | C1QTNF1 | PON1 | KERA |  |  |  |  |  |  |  |  |  |  |  |  |  |  |  |  |
| **P3/B1** | Wnt3 | WFDC13 | LYNX1 | AZGP1 |  |  |  |  |  |  |  |  |  |  |  |  |  |  |  |  |
| **P14/C6** | Wnt3 | IL25 | WFDC7 | KLK15 |  |  |  |  |  |  |  |  |  |  |  |  |  |  |  |  |
| **P6/A2** | Wnt3 | IL27 | WFDC13 | TIMP3 |  |  |  |  |  |  |  |  |  |  |  |  |  |  |  |  |
| **P10/C6** | Wnt3 | DEFB110 | SUMF1 | LCN1 |  |  |  |  |  |  |  |  |  |  |  |  |  |  |  |  |
| **P1/B6** | Wnt3 | REG1A | MASP1 | CCL27 |  |  |  |  |  |  |  |  |  |  |  |  |  |  |  |  |
| **P17/C3** | Wnt8A | DEFB130 | KLK2 | PNLIPRP3 |  |  |  |  |  |  |  |  |  |  |  |  |  |  |  |  |
| **P7/B1** | Wnt10B | WFDC13 | WFDC14 | APOD |  |  |  |  |  |  |  |  |  |  |  |  |  |  |  |  |
| **P12/A1B** | SFTPC | LY86 | Lynx1 | SAA1 |  |  |  |  |  |  |  |  |  |  |  |  |  |  |  |  |
| **P8/B1** | IL21 | WFDC13 | WFDC14 | LCN1 |  |  |  |  |  |  |  |  |  |  |  |  |  |  |  |  |
| **P4/D2** | IL21 | WFDC11 | PRAP1 | SMOC1 |  |  |  |  |  |  |  |  |  |  |  |  |  |  |  |  |
| **P17/B4** | IL21 | TIMP1 | PON1 | NODAL |  |  |  |  |  |  |  |  |  |  |  |  |  |  |  |  |
| **P12/D1** | IL6 | IL20 | WFDC14 | KLK15 |  |  |  |  |  |  |  |  |  |  |  |  |  |  |  |  |
| **P4/C4** | IL27 | WFDC13 | LYNX1 | RCN3 |  |  |  |  |  |  |  |  |  |  |  |  |  |  |  |  |
| **P8/B6** | WFDC13 | PGLYRP1 | PON1 | RCN2 |  |  |  |  |  |  |  |  |  |  |  |  |  |  |  |  |
| **P12/B2** | WFDC13 | PGLYRP1 | STC2 | SRPX |  |  |  |  |  |  |  |  |  |  |  |  |  |  |  |  |
| **P10/A4** | CCL27 | PGLYRP3 | RBP4 | A1BG |  |  |  |  |  |  |  |  |  |  |  |  |  |  |  |  |
|  |  |  |  |  |  |  |  |  |  |  |  |  |  |  |  |  |  |  |  |  |

| **Clones with 5 inserts** | |  |  |  |  |  |  |  |  |  |  |  |  |  |  |  |  |  |  |  |
| --- | --- | --- | --- | --- | --- | --- | --- | --- | --- | --- | --- | --- | --- | --- | --- | --- | --- | --- | --- | --- |
| **Clone label** | **Insert1** | **Insert2** | **Insert3** | **Insert4** | **Insert5** |  |  |  |  |  |  |  |  |  |  |  |  |  |  |  |
| **P15/B6** | IGFBP4 | FGF23 | IL25 | SMOC1 | SCG3 |  |  |  |  |  |  |  |  |  |  |  |  |  |  |  |
| **P10/A6** | IGFBP5 | WFDC14 | TIMP2 | LEAP2 | SDF4 |  |  |  |  |  |  |  |  |  |  |  |  |  |  |  |
| **p6/c2c** | WISP1 | FGF14 | IL22 | BCMP11 | APOD |  |  |  |  |  |  |  |  |  |  |  |  |  |  |  |
| **P9/D2/B** | WISP1 | CTGF | FGFBP1 | IL26 | AHSG |  |  |  |  |  |  |  |  |  |  |  |  |  |  |  |
| **P4/B3** | WISP3 | DKKL1 | CCL23 | KLK2 | KLK14 |  |  |  |  |  |  |  |  |  |  |  |  |  |  |  |
| **P10/C3** | WISP3 | RSPO3 | KLK15 | PON1 | GIF |  |  |  |  |  |  |  |  |  |  |  |  |  |  |  |
| **P16/C3A** | FGFBP2/KSP37 | PON1 | CLEC5A | PTHLH | FMOD |  |  |  |  |  |  |  |  |  |  |  |  |  |  |  |
| **p14/a3b** | Sfrp4 | GDF8 | ADIPOQ | IL6 | DEFB104A |  |  |  |  |  |  |  |  |  |  |  |  |  |  |  |
| **P13/B1** | DKK1 | FGF10 | IL20 | CXCL1 | CXCL13 |  |  |  |  |  |  |  |  |  |  |  |  |  |  |  |
| **P15/C1** | DKK2 | FGF13 | TIMP4 | PON1 | SRPX2 |  |  |  |  |  |  |  |  |  |  |  |  |  |  |  |
| **P1/C2** | DKK4 | FGF4 | TIMP4 | PI15 | PON1 |  |  |  |  |  |  |  |  |  |  |  |  |  |  |  |
| **P12/A6** | RSPO1 | CLEC3A | CCL13 | CST8 | SPARCL1 |  |  |  |  |  |  |  |  |  |  |  |  |  |  |  |
| **P12/C2B** | RSPO3 | FGF1 | IL17F | WFDC4 | NODAL |  |  |  |  |  |  |  |  |  |  |  |  |  |  |  |
| **P13/B3** | FGF12 | IL24 | DEFB106A | PGLYRP3 | LY86 |  |  |  |  |  |  |  |  |  |  |  |  |  |  |  |
| **P2/C1** | FGF13 | TGFB2 | LY86 | TIMP4 | AZU1 |  |  |  |  |  |  |  |  |  |  |  |  |  |  |  |
| **P13/C1** | FGF14 | WFDC4 | wfdc13 | CCL19 | NODAL |  |  |  |  |  |  |  |  |  |  |  |  |  |  |  |
| **P5/D1** | FGF16 | Wnt9a | SFTPC | WFDC13 | INHBA |  |  |  |  |  |  |  |  |  |  |  |  |  |  |  |
| **P13/A2** | Wnt10A | IL6 | AGR2 | DEFB130 | PTHLH |  |  |  |  |  |  |  |  |  |  |  |  |  |  |  |
| **P1/B2** | C1QTNF3 | MDK | WFDC13 | CETP | SAA1 |  |  |  |  |  |  |  |  |  |  |  |  |  |  |  |
| **P12/B4B** | IL9 | IL6 | WFDC4 | WFDC13 | PGLYRP3 |  |  |  |  |  |  |  |  |  |  |  |  |  |  |  |
| **P11/B4** | IL21 | klk2 | PON1 | CLU | SUMF1 |  |  |  |  |  |  |  |  |  |  |  |  |  |  |  |
| **P4/C1** | IL6 | DEFB104A | LYNX1 | TNFAIP6 | RCN2 |  |  |  |  |  |  |  |  |  |  |  |  |  |  |  |
| **P5/A3** | IL27 | WFDC13 | VTN | NODAL | PTHLH |  |  |  |  |  |  |  |  |  |  |  |  |  |  |  |
| **P16/C1** | WFDC13 | PNLIPRP1 | TIMP1 | UTS2 | SUMF1 |  |  |  |  |  |  |  |  |  |  |  |  |  |  |  |
| **P8/A1** | IFNA1 | SFTPC | TIMP3 | NODAL | PTHLH |  |  |  |  |  |  |  |  |  |  |  |  |  |  |  |
| **P7/A1A** | CXCL2 | TIMP3 | AZU1 | SAA1 | APOD |  |  |  |  |  |  |  |  |  |  |  |  |  |  |  |
| **P7/D1** | PGLYRP3 | AZU1 | KLK3 | A1BG | PON1 |  |  |  |  |  |  |  |  |  |  |  |  |  |  |  |
|  |  |  |  |  |  |  |  |  |  |  |  |  |  |  |  |  |  |  |  |  |
| **Clones with 6 inserts** | |  |  |  |  |  |  |  |  |  |  |  |  |  |  |  |  |  |  |  |
| **Clone label** | **Insert1** | **Insert2** | **Insert3** | **Insert4** | **Insert5** | **Insert6** |  |  |  |  |  |  |  |  |  |  |  |  |  |  |
| **P14/B6** | IGFBP5 | FCN3 | MASP1 | VTN | SDF4 | OGN |  |  |  |  |  |  |  |  |  |  |  |  |  |  |
| **P1/B1B** | igfbp-CTGF | MASP2 | WFDC11 | DEFB112 | TIMP2 | SPON2 |  |  |  |  |  |  |  |  |  |  |  |  |  |  |
| **P11/A6/B** | NOV | FGF23 | CXCL9 | PI15 | PON1 | APOH |  |  |  |  |  |  |  |  |  |  |  |  |  |  |
| **P7/A6** | WISP1 | ANGPTL7 | Wnt9A | WFDC13 | IFNA1 | LYNX1 |  |  |  |  |  |  |  |  |  |  |  |  |  |  |
| **P17/B2** | FGFBP2/KSP37 | FGF23 | IL7 | MDK | AZU1 | APOH |  |  |  |  |  |  |  |  |  |  |  |  |  |  |
| **P9/A6** | FGFBP2/KSP37 | Wnt10B | GDDR | REG1A | Wfdc13 | TCN1 |  |  |  |  |  |  |  |  |  |  |  |  |  |  |
| **P9/D4** | CRELD1 | IL9 | IL15 | KLK2 | ORM1 | SUMF1 |  |  |  |  |  |  |  |  |  |  |  |  |  |  |
| **p5/c1b** | FAM3A | Wnt3 | UTS2 | SFTPA2 | PON1 | TCN1 |  |  |  |  |  |  |  |  |  |  |  |  |  |  |
| **P15/A5** | GDF8 | FGF1 | wfdc13 | AZU1 | klk15 | SAA1 |  |  |  |  |  |  |  |  |  |  |  |  |  |  |
| **P17/C5** | GDF8 | EBI3 | WFDC13 | TIMP2 | SRPX | MIA |  |  |  |  |  |  |  |  |  |  |  |  |  |  |
| **P8/C1** | FGF1 | FGF14 | MASP2 | IL9 | UTS2 | SCGB1D1 |  |  |  |  |  |  |  |  |  |  |  |  |  |  |
| **P11/D1A** | FGF1 | FGF11 | WFDC11 | WFDC13 | WFDC14 | RETNLB |  |  |  |  |  |  |  |  |  |  |  |  |  |  |
| **P9/C3** | FGF6 | FGF14 | C1QTNF6 | IL22 | PON1 | PPGB |  |  |  |  |  |  |  |  |  |  |  |  |  |  |
| **P16/C6A** | FGF10 | C1QTNF6 | WFDC13 | CXCL11 | CCL27 | DEFB130 |  |  |  |  |  |  |  |  |  |  |  |  |  |  |
| **P13/D2** | FGF10 | MASP2 | IL17 | IL3 | AGR2 | SCGB1D1 |  |  |  |  |  |  |  |  |  |  |  |  |  |  |
| **P14/C1** | Wnt3 | IL25 | WFDC7 | WFDC13 | KLK15 | PON1 |  |  |  |  |  |  |  |  |  |  |  |  |  |  |
| **P2/C5** | MDK | CXCL13 | KLK2 | SMOC1 | SPON2 | APOH |  |  |  |  |  |  |  |  |  |  |  |  |  |  |
| **P3/B3** | IL12A | IFNA1 | DEFB112 | IHH | RBP4 | RCN2 |  |  |  |  |  |  |  |  |  |  |  |  |  |  |
| **P17/C6** | IL27 | WFDC13 | SAA4 | SCGB3A1 | GREM2 | PTHLH |  |  |  |  |  |  |  |  |  |  |  |  |  |  |

| **Clones with 7 inserts** | | |  |  |  |  |  |  |  |  |  |  |  |  |  |  |  |  |  |  |  |
| --- | --- | --- | --- | --- | --- | --- | --- | --- | --- | --- | --- | --- | --- | --- | --- | --- | --- | --- | --- | --- | --- |
| **Clone label** | **Insert1** | | **Insert2** | **Insert3** | **Insert4** | **Insert5** | **Insert6** | **Insert7** |  |  |  |  |  |  |  |  |  |  |  |  |  |
| **P13/C3** | SOSTDC1 | | PROK2 | FGF4 | IFNE1 | TIMP1 | KLK4 | SUMF1 |  |  |  |  |  |  |  |  |  |  |  |  |  |
| **P10/A2** | DKK2 | | FGF14 | Wnt3 | PGLYRP1 | KLK10 | A1BG | SAA1 |  |  |  |  |  |  |  |  |  |  |  |  |  |
| **P17/C1** | FAM3B | | Wnt3 | IL6 | DEFB114 | BPI | PGLYRP1 | PNLIPRP3 |  |  |  |  |  |  |  |  |  |  |  |  |  |
| **P3/C2** | GDF8 | | CXCL7 | CXCL11 | DEFA6 | TIMP4 | CAMP | PPGB |  |  |  |  |  |  |  |  |  |  |  |  |  |
| **P8/B4** | FGF3 | | Wnt9A | WFDC13 | TIMP4 | PON1 | SMOC1 | APOH |  |  |  |  |  |  |  |  |  |  |  |  |  |
| **P7/D2** | CXCL13 | | PGLYRP3 | LY86 | TIMP3 | LECT2 | PON1 | APOD |  |  |  |  |  |  |  |  |  |  |  |  |  |
|  |  | |  |  |  |  |  |  |  |  |  |  |  |  |  |  |  |  |  |  |  |
| **Clones with 8 inserts** | | |  |  |  |  |  |  |  |  |  |  |  |  |  |  |  |  |  |  |  |
| **Clone label** | **Insert1** | | **Insert2** | **Insert3** | **Insert4** | **Insert5** | **Insert6** | **Insert7** | **Insert8** |  |  |  |  |  |  |  |  |  |  |  |  |
| **P2/B3** | WIF1 | | FGF14 | IL17 | CCL13 | KLK3 | PON1 | SAA1 | LY86 |  |  |  |  |  |  |  |  |  |  |  |  |
| **p9/a5** | wisp3 | | Wnt8a | C1qTNF1 | IL27 | WFDC13 | lynx1 | EBAG9 | PTHLH |  |  |  |  |  |  |  |  |  |  |  |  |
| **P4/A5** | FAM3B | | Wnt8A | REG3G | WFDC13 | RETN | NTF3 | KLK14 | NODAL |  |  |  |  |  |  |  |  |  |  |  |  |
|  |  | |  |  |  |  |  |  |  |  |  |  |  |  |  |  |  |  |  |  |  |
| **Clones with 9 inserts** | | |  |  |  |  |  |  |  |  |  |  |  |  |  |  |  |  |  |  |  |
| **Clone label** | **Insert1** | | **Insert2** | **Insert3** | **Insert4** | **Insert5** | **Insert6** | **Insert7** | **Insert8** | **Insert9** |  |  |  |  |  |  |  |  |  |  |  |
| **P11/A2A** | NOV | | FGFBP2/KSP37 | WFDC4 | NGFB | KLK4 | SMOC1 | LUM | SUMF1 | P4HA2 |  |  |  |  |  |  |  |  |  |  |  |
|  |  | |  |  |  |  |  |  |  |  |  |  |  |  |  |  |  |  |  |  |  |
| **Clones with10 inserts** | | |  |  |  |  |  |  |  |  |  |  |  |  |  |  |  |  |  |  |  |
| **Clone label** | **Insert1** | | **Insert2** | **Insert3** | **Insert4** | **Insert5** | **Insert6** | **Insert7** | **Insert8** | **Insert9** | **Insert10** |  |  |  |  |  |  |  |  |  |  |
| **P16/C4** | IGFBP5 | | RSPO1 | BMP3 | FGF18 | Wnt3 | IL21 | IL12B | PGLYRP1 | TIMP1 | ZG16 |  |  |  |  |  |  |  |  |  |  |
| **P14/C1A** | FGFBP2/KSP37 | | FGF23 | SFTPC | AGR2 | WFDC13 | IFNA1 | TIMP3 | PI15 | NODAL | PTHLH |  |  |  |  |  |  |  |  |  |  |
|  |  | |  |  |  |  |  |  |  |  |  |  |  |  |  |  |  |  |  |  |  |
| **Clones with 11 inserts** | | |  |  |  |  |  |  |  |  |  |  |  |  |  |  |  |  |  |  |  |
| **Clone label** | **Insert1** | | **Insert2** | **Insert3** | **Insert4** | **Insert5** | **Insert6** | **Insert7** | **Insert8** | **Insert9** | **Insert10** | **Insert11** |  |  |  |  |  |  |  |  |  |
| **P14/B2** | FCN3 | | IL9 | EBI3 | IL27 | WFDC5 | WFDC13 | CCL7 | IHH | LYNX1 | F10 | CLEC5A |  |  |  |  |  |  |  |  |  |
|  |  | |  |  |  |  |  |  |  |  |  |  |  |  |  |  |  |  |  |  |  |
|  | |  | | | | | | | | | | | | | | | | | | | |
|  | |  | | | | | | | | | | | | | | | | | | | |
